# Evidence-Based Exercise Recommendations to Improve Mental Wellbeing in Women with Breast Cancer during Active Treatment: A Systematic Review and Meta-Analysis

**DOI:** 10.3390/cancers13020264

**Published:** 2021-01-12

**Authors:** Robinson Ramírez-Vélez, Fabiola Zambom-Ferraresi, Antonio García-Hermoso, Justina Kievisiene, Alona Rauckiene-Michealsson, César Agostinis-Sobrinho

**Affiliations:** 1 Faculty of Health Sciences, Klaipeda University, 92294 Klaipėda, Lithuania; robin640@hotmail.com (R.R.-V.); justina.kievisiene@gmail.com (J.K.); alona.rauckiene-michaelsson@ku.lt (A.R.-M.); 2 Navarrabiomed, Hospital Complex of Navarra (CHN), Navarra Health Research Institute (IdisNa), Public University of Navarra (UPNA), C/Irunlarrea 3, 31008 Pamplona, Spain; fabiolahaole@hotmail.com (F.Z.-F.); antonio.garcia.h@usach.cl (A.G.-H.); 3 Center for Biomedical Research in Network on Healthy Fragility and Aging (CIBERfes), Carlos III Health Institute, 28029 Madrid, Spain; 4 Laboratory of Physical Activity, Sports and Health Sciences, University of Santiago de Chile (USACH), Santiago 71783-5, Chile

**Keywords:** exercise, physical activity, cancer treatment, mental health

## Abstract

**Simple Summary:**

There is uncertainty whether the effects of different types of training (e.g., aerobic, strength, and/or combined) and intensity have different effects on mental health outcomes in women with breast cancer during active treatment. The results showed that exercise training led to a moderate improvement in quality of life by the Functional Assessment of Cancer Therapy-Breast Cancer (FACT-B) instrument and small improvements in mental wellbeing domains, such as anxiety, body image, depression, overall quality of life, and emotional function.

**Abstract:**

Breast cancer was the most common cancer in women worldwide. The aims of the current systematic review and meta-analysis are: (i) to systematically examine the effects of exercise interventions on mental wellbeing; (ii) to examine the specific effect of the type of supervised exercise and its intensity, volume and frequency on mental wellbeing; and (iii) to explore which interventions are most effective in mental wellbeing among women with breast cancer during active treatment. An electronic literature search was performed using MEDLINE (via PubMed), Embase (Ovid), and Web of Science, we identified 175 full-text articles. The 57 publications included data from 6988 participants, age ranging from 18 to 78 years (weighted mean: 50.85 years). Compared with the control conditions, exercise training programs were associated with significant reductions in anxiety (d = −0.22, I^2^ = 53.0%), depression (d = −0.24, I^2^ = 66.6%), and fatigue (d = −0.47, I^2^ = 69.8%), as well as increases in body image (d = 0.27, I^2^ = 69.2%) and quality of life (overall, d = 0.46, I^2^ = 71.6%; emotional function, d = 0.33, I^2^ = 65.7%; and FACT-B, d = 0.60, I^2^ = 76.2%). There were a variety of frequencies, intensities, and durations of supervised exercise programs reported in the included meta-analytic approach. In addition, we found that concomitant concurrent training, at moderate-to-vigorous intensity, and with a volume ≥50 min/week had benefits on a number of health outcomes, such as fatigue, depression, and quality of life measure by the FACT-B instrument. These findings have important implications for healthcare providers and multidisciplinary teams involved in mental health management in cancer patients during active treatment.

## 1. Introduction

Breast cancer was the most common cancer in women worldwide, with an estimated 268,600 newly diagnosed women with invasive disease according to the National Cancer Institute and Surveillance, Epidemiology, and End Results Program (in 2019), accounting for approximately 15–30% of all new cancer cases among women, depending on the data sources [1,2]. Over 1.5 million women (25% of all women with cancer) are diagnosed with breast cancer every year throughout the world [2]. Each year, nearly 42,000 women die of breast cancer, making it the second-leading cause of cancer deaths among US women after lung cancer [3].

Dramatic changes in lifestyle and biologic mechanism, including high rates of physical inactivity, excess body fat and sedentary time, age at menarche and fertility, food environment, family history, gene mutations, sex steroids and metabolic hormones, insulin sensitivity, and chronic inflammation contribute substantially to the development of non-communicable diseases, including cancer [4]. Women treated for breast cancer frequently experience numerous disease- or treatment-related adverse outcomes (physiologic, psychosocial, or both) and poorer mental wellbeing. Side effects that appear with adjuvant cancer treatment differ depending on the mode of treatment, which is radiotherapy, chemotherapy, hormonal, or antibody therapy [5]. In addition, these unwanted effects can be prolonged after completion of active treatment and may hinder the woman’s return to normal life [6]. There is an increasing need for rehabilitation to address these issues.

Exercise interventions may be effective in managing some of these side effects, such as fatigue, depression, and cognitive dysfunction [7]. In particular, supervised exercise represents a modifiable health behavior that could alleviate the sequelae related to breast cancer and assist women in returning to the health status they had before receiving the diagnosis and treatment [8]. Despite these benefits of exercise, there is a lack of evidence on the safety and efficacy of exercise in relation to dose [9]. Current recommendations for cancer patients [10] are to avoid inactivity, return to normal daily activities as quickly as possible after surgery, continue these activities during and after non-surgical treatments, and engage in 150 min of moderate intensity aerobic activity per week [11] or resistance exercise (i.e., strength training exercises at least 2 days per week).

Recent reviews have reported a low number of serious adverse effects were reported amongst the studies, providing reassurance that exercise is safe for this population [7,12,13]. In this line, evidence supporting the effect of supervised exercise interventions on improved mental wellbeing is limited in women treated for breast cancer. Further complicating the ability to harmonize information around exercise prescription is the variability across studies regarding exercise type (i.e., aerobic, resistance, or combined exercise training), intensity (i.e., moderate or vigorous), volume and frequency of exercise needed for exercise prescription [14].

There is currently a lack of high-quality evidence to support optimal dose to women treated for breast cancer according to an individual’s characteristics or other treatment effect modifiers. The most effective type, duration and intensity of exercise remains unclear. Prior meta-analyses have not adequately addressed these questions as many have not been breast cancer-specific, and many have had design limitations such as relying on mental wellbeing scores to evaluate intervention efficacy and/or focusing on individual modes of intervention. As such, it is imperative to improve the recommendations of existing guidelines and to inform decision makers about the safety and effectiveness of these interventions.

Therefore, the aims of the current systematic review and meta-analysis are: (i) to systematically examine the effects of exercise interventions on mental wellbeing (i.e., mood states, anxiety, depression, quality of life, self-esteem, or fatigue) in women with breast cancer; (ii) to examine the specific effect of the type of supervised exercise (aerobic, resistance or combined exercise training) and its intensity (i.e., moderate or vigorous), volume and frequency on mental wellbeing in women with breast cancer; and (iii) to explore which interventions are most effective in mental wellbeing among women with breast cancer during active treatment.

## 2. Materials and Methods

The study was conducted and reported according to the Preferred Reporting Items for Systematic Reviews and Meta-Analyses [15] guidelines and is awaiting registration in the PROSPERO International Prospective Register of Systematic Reviews. The entire process from literature selection to data extraction was performed independently by two authors (R.R.-V. & F.Z.-F.). Any disagreements were resolved through consultation with a third researcher (A.G.-H.).

### 2.1. Search Strategy

Three electronic databases, including MEDLINE (via PubMed), Embase (Ovid), and Web of Science, were searched in June 2020. The database searches were supplemented by citation tracking of the included articles using Google Scholar and checking the reference lists of the included studies. The search strategy incorporated the recommendations for a highly sensitive search strategy for the retrieval of clinical trials on PubMed [16]. The research strategy followed the following related format: randomized controlled trial, breast cancer, exercise and mental wellbeing (randomized controlled trial (or controlled clinical trial or “randomi*” or trial or clinical trials) and breast and cancer or (neoplasm or “tumour*” or “tumor*” or “carcino*” or “leukaemi*” or “leukemi*”) and physical activity or exercise (aerobic or endurance or resistance or strength or flexibility or stretching) and mental wellbeing or mental health or (mood states or anxiety or depression or “quality of life” or self-esteem or fatigue). The full detailed research strategy is described in Table S1.

### 2.2. Eligibility Criteria

Two authors (F.Z.-F. and R.R.-V.) independently reviewed all the retrieved studies against the inclusion criteria (Table 1). The title and abstract were examined, and full texts were obtained if there was ambiguity regarding eligibility. Disagreements were resolved by discussion, with a third reviewer consulted when necessary (A.G.-H.). Additionally, the reference lists of relevant systematic reviews and meta-analyses were also examined to identify additional studies.

To identify relevant randomized controlled trials (RCTs), the search was based on predefined terms regarding population, intervention, comparison, and outcome (PICO terms) using both MeSH terms and free text: Population (P): women with breast cancer who are undergoing (neo-)adjuvant cancer treatment (hormonal therapy, chemotherapy, radiotherapy, mastectomy or combination); Intervention (I): physical exercise or home exercise interventions had to meet the definition of “physical activity that is planned, structured and repetitive and has a final or intermediate objective of the improvement or maintenance of physical fitness” [17] with aerobic/endurance, stretching/flexibility, resistance/strengthening or combined training as a key component, because these modes of exercise are expected to result in significant physiological changes; Comparison (C): women receiving the standard of care or who were on a waiting list or on attention control; and Outcome (O): reported at least one of the primary outcomes (i.e., mood states, anxiety, depression, quality of life, self-esteem, or fatigue). The classification of prescribed exercise intensity was based on the American College of Sports Medicine (ACSM) guidelines, including the timing and mode of intervention delivery, intervention duration, and exercise dimensions, in terms of frequency, intensity, time and type (FITT factors), plus duration (D) of the intervention [10]. All the included studies were published in English.

### 2.3. Data Extraction

Reviewers (F.Z.-F. and R.R.-V.) independently extracted the following information regarding the study population: first author (year), disease stage (breast cancer description), (neo-)adjuvant cancer treatment timing, number of participants, age, prescribed exercise intervention according to FITT-D, and primary outcomes (measure instrument/tool). For studies using a combined exercise intervention (i.e., aerobic and resistance training and/or stretching/flexibility), the intensity or total duration for both components must have been specified. Furthermore, the intensity (e.g., percentage of maximum heart rate, repetition maximum etc.) or duration of completed exercise needed to be reported.

### 2.4. Risk of Bias and Quality Assessment within Studies

The methodological quality of the studies, including their risk of bias was assessed using the Physiotherapy Evidence Database (PEDro) [18] scale. The PEDro scale rates the methodological quality of randomized trials out of 10. Scores were based on all the information available from the published version. A score of 5 out of 10 was set as the minimum score for inclusion in the review. The quality assessments of the reviewers were compared, and disagreements were resolved by discussion among all four raters.

### 2.5. Statistical Analysis

All analyses were conducted using the random-effects inverse-variance model with the Hartung-Knapp-Sidik-Jonkman adjustment and carried out using STATA software (version 16.1; StataCorp, College Station, TX, USA). Changes in mental wellbeing parameters for RCTs were calculated using Cohen’s d by subtracting change differences between the exercise and control groups, using the pooled standard deviation (SD) of change in both groups. If change scores SD were not available, they were calculated from 95% confidence intervals (CIs) for either change outcome or exercise training effect differences, as well as pre-SD and post-SD values [19]. Qualitative descriptors used to interpret the strength of the SMDs were based on Cohen’s [20] criteria: trivial (<0.2), small (0.2 to 0.49), moderate (0.5 to 0.79) and large (≥0.8). Heterogeneity across RCTs was calculated using the inconsistency index (I^2^) [21].

A subgroup analysis was conducted according to type of exercise training (aerobic, strength, and concurrent), meeting or not meeting the World Health Organization’s recommendations for physical activity (moderate levels < 150 min/week and ≥150 min/week) and exercise intensity using the categorization of the ACSM [22] (moderate, moderate-to-vigorous and vigorous intensity). Random-effects meta-regression analyses using method of moments (DerSimonian and Laird method) were also estimated to evaluate whether results were different by duration of the interventions (months).

Small-study effects and publication bias were examined using the Doi plot and Luis Furuya-Kanamori (LFK) index, both of which have been shown to be superior to the traditional funnel plot and Egger’s regression intercept test [23]. Values of −1, between −1 and −2, and >−2, are considered to represent no, minor, and major asymmetry, respectively [24].

## 3. Results

### 3.1. Study Selection

**Figure 1:**
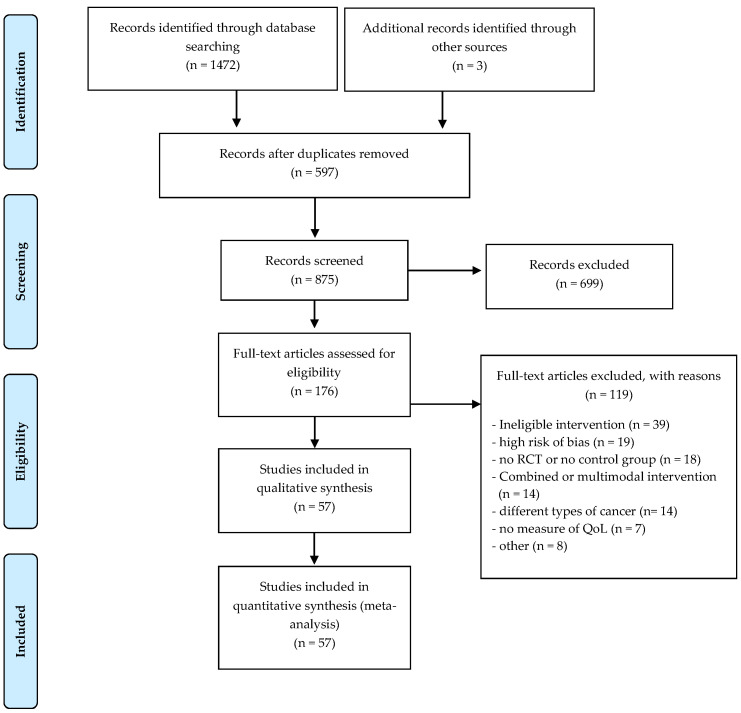
PRISMA flowchart of studies through the review.

Through database searching of MEDLINE (via PubMed), Embase (Ovid), and Web of Science, we identified 1472 records. Of these, 597 studies were excluded by duplication. We screened the titles and abstracts of 875 records, excluding 699 records. We screened 176 full-text articles. The 57 articles that were eligible for the review were supplemented by three more identified through the reference lists and other additional sources. Therefore, we included a total of 57 [25,26,27,28,29,30,31,32,33,34,35,36,37,38,39,40,41,42,43,44,45,46,47,48,49,50,51,52,53,54,55,56,57,58,59,60,61,62,63,64,65,66,67,68,69,70,71,72,73,74,75,76,77,78,79,80,81] studies in the review. The selection process of relevant studies retrieved from databases is shown in a PRISMA-compliant flow chart (Figure 1).

### 3.2. Risk of Bias and Quality Assessment within Studies

The assessment of risk of bias showed a mean PEDro score of 6.6 (SD 1.1), indicating consistent methodological quality and a low risk of most biases, except blinding. Quality ratings for each study are presented in Table S2.

### 3.3. Characteristics of Included Trials

The methodological details of the included studies are presented in Table 2, characteristics of included studies. The characteristics of study populations, intervention protocols and outcome measures and tools of measure are briefly described. All 57 selected studies, all RCTs in their design and published between 2001 and 2020 [25,26,27,28,29,30,31,32,33,34,35,36,37,38,39,40,41,42,43,44,45,46,47,48,49,50,51,52,53,54,55,56,57,58,59,60,61,62,63,64,65,66,67,68,69,70,71,72,73,74,75,76,77,78,79,80,81], provided enough information to be included in the meta-analysis.

### 3.4. Participants

The 57 publications included data from 6988 participants, age ranging from 18 to 78 years (weighted mean: 50–85 years). Sample sizes ranged from 18 to 500 individuals.

### 3.5. Interventions

Interventions included aerobic exercise (19); resistance exercise (11); a combination of both (21); an arm with aerobic exercise and another of resistance training (6); or a multicomponent program combining aerobic exercise, strength training, and flexibility (3); and additionally, one intervention of resistance exercise and impact training, one of resistance and flexibility and a placebo exercise intervention (only flexibility exercise). The mean exercise frequency was 3.2 sessions/week (range: 1–6 sessions/week). The duration of the interventions varied from 6 to 52 weeks (mean: 17.8 weeks). Among the 57 studies analyzed, we observed 58 interventions, including the following five different types: supervised exercise (44), home-based (with follow-up) (14), combined (those beginning supervised and finishing self-administered) (14), mixed (with one intervention group supervised and another home-based) (3), and interventions supervised via Internet/app (2).

### 3.6. Outcome Measures

**Figure 2:**
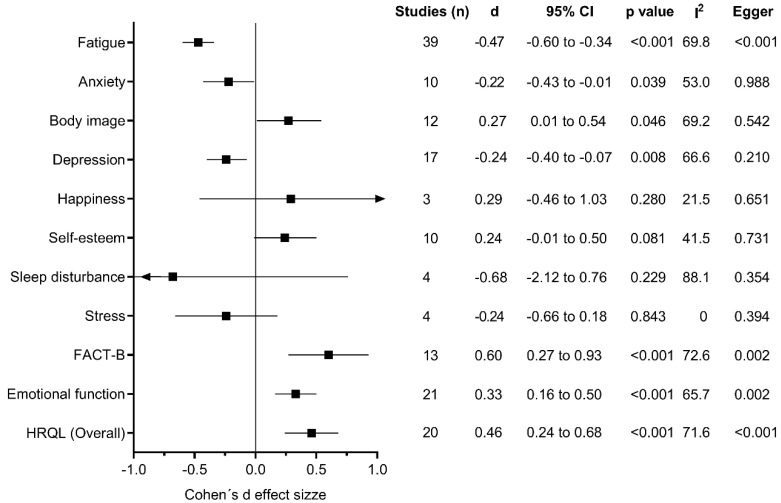
Exercise effects on mental health parameters.

Primary outcomes included and pooled in the present meta-analysis were anxiety, body image, depression, fatigue, happiness, quality of life (overall, emotional function, and FACT-B total score), stress, self-esteem, and sleep disturbance (Figure 2).

### 3.7. Summary of Evidence and Heterogeneity for Meta-Analysis

Compared with the control conditions, exercise training programs were associated with significant reductions in anxiety (d = −0.22, 95% CI −0.43 to −0.01; I^2^ = 53.0%), depression (d = −0.24, 95% CI −0.40 to −0.07; I^2^ = 66.6%), and fatigue (d = −0.47, 95% CI −0.60 to −0.34; I^2^ = 69.8%), as well as increases in body image (d = 0.27, 95% CI 0.01 to 0.54; I^2^ = 69.2%) and quality of life (overall, d = 0.46, 95% CI 0.24 to 0.68; I^2^ = 71.6%; emotional function d = 0.33, 95% CI 0.16 to 0.50; I^2^ = 65.7%; and FACT-B, d = 0.60, 95% CI 0.27 to 0.93; I^2^ = 76.2%). There was evidence of large heterogeneity for quality of life overall (I^2^ = 71.6%, *p* < 0.001) and moderate heterogeneity for measures of emotional function (I^2^ = 65.7%, *p* < 0.001), and fatigue (I^2^ = 69.8%, *p* < 0.001) (Figure 2 and Table S3).

### 3.8. Subgroup Analysis

**Figure 3:**
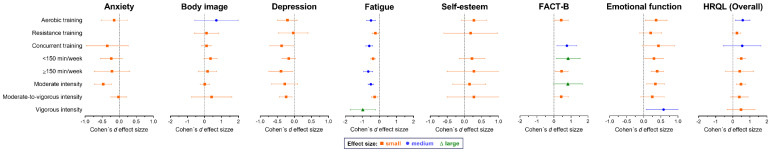
Exercise effects on mental health parameters.

Results according to the characteristics of the exercise programs are shown in Figure 3 and Table S4 and Figures S5–S15. Concurrent training programs seem to favor higher effects on depression (d = −0.37, 95% CI −0.72 to −0.02; I^2^ = 70.7%), fatigue (d = −0.59, 95% CI −0.79 to −0.38; I^2^ = 62.5%), and quality of life assessed with the FACT-B questionnaire (d = 0.76, 95% CI 0.18 to 1.35; I^2^ = 83.0%). Also, exercise programs that met ≥150 min of physical activity per week favored higher reductions in fatigue (d = −0.65, 95% CI −0.93 to −0.38; I^2^ = 76.3%) and depression (d = −0.39, 95% CI −0.75 to −0.04; I^2^ = 50.7%). Regarding intensity, exercise programs at vigorous intensity favored further reductions in fatigue (d = −0.97, 95% CI −1.70 to −0.24; I^2^ = 71.7%) and an increase in emotional function (d = 0.58, 95% CI 0.07 to 1.09; I^2^ = 0%). However, moderate intensity training also favored significant effects on anxiety (d = −0.47, 95% CI −0.72 to −0.22; I^2^ = 0%), fatigue (d = −0.50, 95% CI −0.66 to −0.34; I^2^ = 48.0%), and quality of life (overall, d =0.48, 95% CI 0.20 to 0.76; I^2^ = 0% and emotional function, d = 0.34, 95% CI 0.08 to 0.61; I^2^ = 41.7%).

### 3.9. Meta-Regression Analysis

Based on meta-regression analyses, there were significant effects of duration of the intervention on the effect size estimate for overall quality of life (β = −0.023; *p* = 0.009) and FACT-B (β = −0.049; *p* = 0.029), but not for anxiety (β = 0.018; *p* = 0.306), body image (β = 0.014; *p* = 0.081), depression (β = 0.004; *p* = 0.590), happiness (β = −0.044; *p* = 0.613), self-esteem (β = −0.021; *p* = 0.363), sleep disturbance (β = −0.084; *p* = 0.249), stress (β = 0.020; *p* = 0.315), and emotional function (β = −0.013; *p* = 0.120) (Figures S16–S26).

### 3.10. Publication Bias

Overall, no asymmetry suggestive of small-study effects was observed except for depression (LKF index = −2.24), emotional function (LKF index = 2.87), and overall quality of life (LKF index = 2.83) (Figures S27–S37).

## 4. Discussion

This is the first meta-analytic approach to quantitatively synthesize the effects of supervised exercise interventions on mental wellbeing in women with breast cancer during active treatment. The results showed that exercise training led to a moderate improvement in quality of life by the Functional Assessment of Cancer Therapy-Breast Cancer (FACT-B) instrument and small improvements in mental wellbeing domains, such as anxiety, body image, depression, overall quality of life, and emotional function. Furthermore, the *I*² range was 53.0% to 76.2%, suggesting moderate to substantial heterogeneity. There were a variety of frequencies, intensities, and durations of supervised exercise programs reported in the included meta-analytic approach. For example, we found that concomitant concurrent training, at moderate-to-vigorous intensity, and with a volume ≥150 min/week had benefits on a number of health outcomes, such as fatigue, depression, and quality of life measure by the FACT-B instrument. These findings have important implications for healthcare providers and multidisciplinary teams involved in mental health management in cancer patients during active treatment.

Regarding evidence-based exercise recommendations, this is the first systematic review and meta-analysis providing evidence of the efficacy of the type, intensity, and volume of supervised exercise interventions on mental wellbeing in women with breast cancer. Health-related quality of life, which was assessed using the Medical Outcomes Study Short Form (MOS SF-36), World Health Organization Quality of Life-BREF (WHOQOL-BREF), European Organization for Research and Treatment of Cancer Quality of Life Questionnaire (EORTC QLQ-C30), FACT-B instrument, and emotional domain in the MOS SF-36 item, had a moderate effect size and substantial heterogeneity between studies. As for the other characteristics of the supervised exercise programs, there were diverse findings across outcomes. The duration of interventions ranged from 6 to 52 weeks across all the RCTs. A large range of supervised exercise intensities (i.e., resistance training 30–90% 1RM and aerobic training 30–90% HR_max_/HRR/VO_2 max_) were reported in the included studies. Moderate intensity plus aerobic exercise training was associated with improvements in overall quality of life (values ranged from 0.48 to 0.58 Cohen’s *d*), while concurrent training at moderate-to-vigorous intensity with a volume ≥ 150 min/week was associated with a moderate to large effect improvement in the functional domain measured by the FACT-B instrument. Furthermore, the three RCTs [36,76,80] employing aerobic programs alone with low-volume (<150 min/week) were also associated with large effect size improvements in overall quality of life (values ranged from 1.29 to 1.89 Cohen’s *d*), and concurrent programs were associated with an improvement in the functional domain measured by the FACT-B instrument (values ranged from 0.52 to 2.06 Cohen’s *d*). Emotional function, as measured by the MOS SF-36 item, showed a moderate effect size (*d* = −0.33, 95% CI −0.16 to −0.50) with moderate heterogeneity across studies (I^2^ = 65.7%). A consistent feature of exercise interventions was that the activities required a moderate-to-vigorous degree of effort, which was consistent with the current recommendations for cancer patients [10] that recommend 150 min of moderate intensity aerobic activity per week [11] or resistance exercise (i.e., strength training exercises at least 2 days per week). Further RCTs involving exercise interventions during adjuvant therapy with larger sample sizes could be conducted to minimize heterogeneity across studies.

In addition, we showed that concurrent training had a moderate positive effect on fatigue in women with breast cancer during active treatment (d = −0.59, 95% CI −0.79 to −0.38; I^2^ = 62.5%). Furthermore, the eight trials employing different forms of intervention combining aerobic plus resistance interventions and aerobic or resistance training alone with a volume ≥150 min/week [32,33,40,41,46,47,51,67] demonstrated significant effect sizes across various fatigue scales. This is an important finding, because the beneficial effects of exercise interventions for both patients in active treatment (chemotherapy or radiation) and breast cancer survivors have been previously reported [82,83,84,85]. For instance, Kidwell et al. [86] suggest that patients with more fatigue at the initiation of adjuvant endocrine therapy are more likely to discontinue its use prematurely, and Giese-Davis et al. [87] describe that cancer-related fatigue is associated with poorer treatment response, progression-free survival, and overall survival. These findings could help to provide more specific exercise recommendations for the management of cancer-related fatigue in women with breast cancer during active treatment.

The incidence of depression in breast cancer has also increased, with rates as high as 17–50% in some [88,89] cross-sectional and prospective observational studies. However, physical exercise may have the potential to reduce depressive symptoms and improve cognition in patients with breast cancer [90]. In this context, our results show that combining the aerobic and resistance interventions has beneficial effects on depression as compared to the control group (d= −0.37, 95% CI −0.72 to −0.02; I^2^ = 70.7%). Furthermore, the three studies employing different forms of intervention combining aerobic plus resistance interventions and aerobic with a volume ≥ 150 min/week [40,41,67] and two studies employing similar forms of concurrent training with a volume < 150 min/week [34,35,36,37,38,39,40,41,42,43,44,45,46,47,48,49,50,51,52,53,54,55,56,57,58,59,60,61,62,63,64,65,66,67,68,69] demonstrated small to large effect sizes across the depression outcome. This finding is consistent with a recently published systematic review and meta-analysis that showed exercise significantly reduced depression in patients with breast cancer [91]. However, our findings suggest that more studies are needed to determine the optimal forms of exercise to ameliorate the depression levels.

Anxiety as measured by the 7-item Social Physique Anxiety Scale (SPAS-7), Hospital Anxiety and Depression Scale (HADS), Hospital Anxiety and Depression Scale-Anxiety (HADS-A), Hospital Anxiety and Depression Scale-Depression (HADS-D), Spielberger’s State Anxiety Inventory (SAI), State-Trait Anxiety Inventory (STAI), and State-Trait Anxiety Index-State Anxiety (STAI-YI) and body image as measured by the Body Image Scale (BIS) and Body Esteem Scale (BES) had small effect sizes (values ranged from −0.22 to 0.27 Cohen’s *d*) and moderate heterogeneity between studies (I^2^ range 53.0–69.2%). This is likely due to differences in intervention characteristics (e.g., exercise type, duration, training load and training volume) and methodologies used to assess mental outcomes. However, these findings suggest that several forms of supervised exercise interventions could ameliorate the various dimensions of anxiety and body image.

The effectiveness of exercise interventions on happiness, self-esteem, sleep disturbance and stress outcomes were analyzed. As would be expected when combining the results of disparate intervention formats with different proposed mechanisms of action, there was considerable heterogeneity observed across alternative intervention types (I^2^ range was 0–88.1%). For example, one study reported a significant large effect of aerobic training with a volume ≥ 150 min/week (d = −1.84, 95% CI −2.36 to −1.32) on sleep disturbance [47], while the other study [80]. With a volume <150 min/week, also observed evidence of a substantial effect (d = −0.80, 95% CI −1.28 to −0.32). Thus, these results suggest that the design of future supervised exercise interventions in women with breast cancer during active treatment should be taken with caution given the limited number of studies included in the analysis. Even so, more data from RCTs are required to substantiate the effect of exercise training on happiness, self-esteem, sleep disturbance and stress outcomes.

Exercise interventions could influence positive effects in cancer patients during active treatment by several potential mechanisms. First, exercise enhances immune function and decreases inflammatory factors [92]. Cancer patients who participated in exercise had increased immune cell activity, such as CD3+ T lymphocytes, including CD4+ and CD8+, αβ T cells, γδ T cells, CD3−/CD16+/56+ NK cells and CD19+ B cells [93] and decreased insulin-like growth factors [94] and cytokines [95], which are probable causes of carcinogenesis. Second, increased participation in exercise interventions helps to maintain physical fitness, including body composition and psychological benefits, such as cardiovascular function and metabolic parameters. Third, cancer patients participating in regular exercise during adjuvant therapy felt less pain and symptoms of emotional-related distress. Mood elevation after exercise is probably also modulated by other neural factors and neurotransmitter systems, such as the endocannabinoid system [96]; nevertheless, evidence-based studies in women with breast cancer need to be conducted.

Limitations of this meta-analytic approach include the lack of evidence for some important outcome measures, methodological limitations of the RCTs, and a high degree of heterogeneity among the trials, in part due to the differences in inclusion criteria and type, intensity, volume, frequency, and duration of the exercise interventions. Averaging did not allow for the presentation of detailed characteristics for the exercise interventions. Curiously, all trials in this meta-analytic approach were classified as having good methodological quality among 11 mental health conditions. However, these estimates generally indicated marked effects, so clinical implications remain clear despite that uncertainty. In addition, the systematic search was limited to English-language manuscripts available in full text and therefore may have missed some relevant trials. Despite these limitations, several strengths need to be acknowledged in this study. For this systematic meta-analytic approach, many different reference databases were used. Furthermore, we were able to include meta-analysis and subgroup analysis that may help to clarify the overall effects of exercise interventions, as well as the specific effects of the different type, intensity, frequency, and volume in women with breast cancer during active treatment.

## 5. Conclusions

To conclude, this review showed that, in women with breast cancer, supervised exercise interventions during adjuvant therapy had a moderate positive effect on quality of life per the FACT-B scale and small improvements in mental wellbeing domains, such as anxiety, body image, depression, overall quality of life, and emotional function. In contrast, the interval estimates for the effect of exercise training on happiness, self-esteem, sleep disturbance and stress levels shows a high level of uncertainty, primarily due to the low number of studies and/or intervention characteristics (e.g., exercise type, duration, training load and training volume).

Oncologists, cancer nurses, physiotherapists, and exercise specialists can refer to the recommendations of this meta-analysis: the average exercise intervention that had a beneficial effect in breast cancer patients during adjuvant therapy was moderate-to-vigorous (i.e., resistance training 30–90% 1RM and aerobic training 30–90% HR_max_/HRR/VO_2 max_) aerobic, resistance, or concurrent exercise for 150 min, 2 to 3 times per week. The intervention of these above-mentioned specialists is probably necessary at different time points to provide the best care. Also, comprehensive cancer centers with exercise-oncology units are neccesary to implement these programs in a timely and organized manner. Further studies are needed with rigorous methodology design and greater sample sizes that take into consideration different methods to study the mental wellbeing effects of exercise interventions. These programs should include clear and detailed presentation of the exercise protocol and its monitoring (i.e., type, intensity, adherence, compliance).

## Figures and Tables

**Table 1:** Inclusion criteria according to PICO format.

**Design**
Randomized controlled trials, low risk bias (A score of 5 out of 10).
**Population**
Breast cancer survivors (>18 years old) without restriction to stage of disease or undergoing (neo-)adjuvant cancer treatment (hormonal therapy, chemotherapy, radiotherapy, mastectomy, or combination).
**Intervention**
*Exercise programs:*
—Aerobic or endurance exercise
Strengthening or resistance training
Stretching
Combined exercise training (i.e., aerobic plus resistance training and/or stretching training)
**Comparisons**
Supervised exercise or home exercise versus women receiving standard of care
Supervised versus or home exercise women who were on a waiting list or on attention control (where reported)
**Outcome Measures**
*Mental wellbeing or mental health:*
Quality of life (overall, FACT-B, emotional functions)
Fatigue
Self-esteem
Depression
Anxiety
Sleep
Body image

**Table 2:** Characteristics of the studies includes.

Study (Year) [Reference] Acronym	BC Stage Treatment Timing	Groups Sample Size	Age	WHO PA Guidelines Supervised or Home-Based		F Ses/ Wk	I	T Min/ Ses	T	D	Outcome Measures	PRO Measure Instrument
Adams (2016) [25] START	I–III a (During CT)	*N* = 200 AE = 64 RE = 66 UC = 70	48.8 (25–78)	🗹 AE 🗹 RE	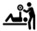 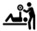	3	Moderate- vigorous 60–80% VO_2_ peak 2 × 8–12 reps (9 exercises) 60–70% 1RM	60′	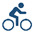 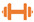	17 (9–24)	QoL, Fatigue	FACT-Na, TOI-An subscale, Fatigue subscale
Ammitzboll (2019) [26] LYCA	I–III (CT, RT, HT; First year after BC surgery)	*N* = 158 RE = 82 UC = 76	53 (33–73) 52 (30–74)	🗹 RE	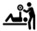 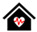	3	Low-moderate 3 × 8–12 reps (6 exercises)	50–55′	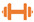	52	HRQoL, Fatigue	EORTC QLQ C30, FACIT, EORTC C30, subscales
Baglia (2019) [27] HOPE	I–III (CT/RT)	*N* = 121 Exc = 61 UC = 60	62.0 (7.0) 60.5 (7.0)	🗹 AE + RE	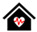 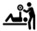	3-5 (AE) 2 (RE)	50–80% HRmax	150′ (AE)	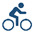 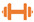	12	QoL, Fatigue	FACT, FACT-G, FACT-B, SF-36, FACIT-Fatigue
Basen- Engquist (2020) [28]	II–III (CT/ RT—during CT treatment)	*N* = 37 Exp = 19 UC = 18	49.6 (13.3) 49.2 (9.2)	🗹 RE + Flexibility exercises	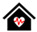	3	Moderate	60′	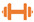 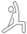	13	QoL, Body Image	SF-36, BIS
Bloomquist (2019) [29]	I–III (CT—during treatment)	*N* = 153 Exc = 75 Con = 78	51.5 (9.6) 52.0 (9.3)	🗹 HIGH RE + AE 🗵 Con = AE	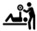 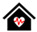 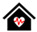	3	70–90% 1 RM (RE) 85–90% HRmax (HIIT) Low-intensity (target = 10,000 steps)	90–120′	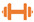 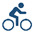 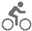	12	QoL	EORTC QLQ- BR23
Cadmus (2009) [30] IMPACT	0–III a (CH/RT/HT—during treatment)	*N* = 50 Exc = 25 UC = 25	54.5 (8.2) 54.0 (10.9)	🗹 AE	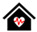	5	Moderate-vigorous (60–80% HRmax)	30′	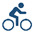	24	Happiness depression anxiety, stress, self-esteem, QoL	HM CES-D STAI, 10-PSS Rosenberg S-ES, FACT-B SF-36
Cadmus (2009) [31] YES	0–III a (CH/RT/HT—Post-treatment, 6-moth follow-up)	*N* = 75 Exc = 37 UC = 38	56.5 (9.5) 55.1 (7.7)	🗹 AE	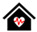	5	Modrate-vigorous (60–80% HRmax)	30′	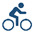	24	Happines, Depression, Anxiety, Stress, Self-esteem, QoL	HM, CES-D, STAI-YI, 10-PSS, Rosenberg S-ES, FACT-B SF-36
Campbell (2005) [31]	I–II (CT/RT—during treatment)	*N* =22 Exc = 12 UC = 10	48 (10) 47 (5)	🗵 AE + RE	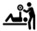	2	60–75% HRmax	20′	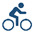 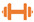	12	Fatigue QoL	FACT-G FACT-B SWLS, PFS
Cantarero-Villanueva (2011) [32] CUIDATE	I–III A (CT/RT—Post-treatment)	*N* = 67 Exc = 32 UC = 35	49 (9) 48 (9)	🗵 Multimodal intervention	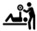	3	Low-intensity	90′	-	8	Fatigue	PFS
Cantarero-Villanueva (2013) [33]	I–III A (CT/RT—Post-treatment)	*N* = 61 Exc = 32 UC= 29	49 (7) 47 (8)	🗹 AE + RE (deep pool)	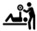	3	60–75% HRmax	60′	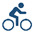 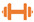	8	Fatigue Mood state	PFS Profile of Mood States (Spanish version)
Carayol (2019) [34] APAD1	I–III c (CT/RT—during treatment)	*N* = 143 Exc = 72 UC = 71	51.2 (10.9) 52.1 (9.3)	🗵 RE + AE + nutritional therapeutic education sessions	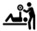	2 (AE) 1 (RE)	Moderate (AE) 50–75% HRmax	30–45′	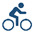 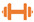	26′	Fatigue QoL Anxiety Depression	MFI, EORTC QLQ-C30 HADS
Casla (2015) [35]	I–III (CT/RT—post-treatment)	*N* = 94 Exc = 47 UC = 47	45.91 (8.21) 51.87 (8.21)	🗹 AE + RE	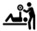	3	Progresive 55–85% HRR (AE) 10–20 Borg Scale (RE)	60′	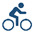 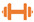	12	QoL	SF-36
Cormie (2013) [36]	I–III (CT/RT/HT—timing not reported)	*N* = 62 Exc HL = 22 Exc LL = 21 UC= 19	56.1 (8.1) 57.0 (10.0) 58.6 (6.7)	🗹 RE HL = High-load 🗹 RE LL = Low-load	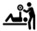 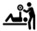	2	Moderate-high 12–16 Borg scale [RPE] RE HL (10–6 reps, 75–85% 1RM); RE LL (20–15 reps, 55–65% 1RM)	60′	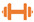 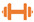	12	QoL	QLQ-BR23 SF-36
Cornette (2016) [37] SAPA	I–III b (CT/RT—during treatment)	*N* = 42 Exc = 20 UC = 22	52 (37–73) 49 (37–68)	🗵 AE (APA) + RE	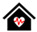	2 (AE) 1 (RE)	60 cycling revolutions/min or HR RE (2 sets of 8–12 reps of 5 exercises)	25-45′	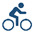 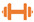	27	QoL, Fatigue, Anxiety and Depression	MFI-20, EORTC QLQ-C30, HADS
Courneya (2003) [38]	I–III a (CH/RT/HT—post-treatment)	*N* = 52 Exc = 24 UC = 28	59 (5) 58 (6)	🗵 AE	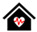	3	70–75% VO_2_ max	35′	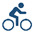	15	QoL, Well-being, Happiness, Self-Esteem, Fatigue	FACT-B, FACT-G, HM, Rosenberg S-ES, FACT-F
Courneya (2007) [39]	I–III a (CT—during adjuvant CT)	*N* = 242 Exc AE = 78 Exc RE = 82 UC = 82	49.2 (25–78) 49.0 (30–75) 49.5 (25–76) 49.0 (26–78)	🗵 AE RE	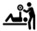	3	60–80% VO_2_ max 60–70% 1RM	15–45′	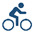 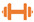	17 (9–24)	Fatigue, Self-Esteem, Depression, Anxiety	FACT-A, Rosenberg S-ES, CES-D, Spielberger SAI
Daley (2007) [40]	Stage not reported (CT/RT/HT—post-treatment)	*N* = 108 Exc = 34 Exc PE = 36 UC = 38	51.6 (8.8) 50.6 (8.7) 51.1 (8.6)	🗹 AE	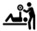	3	Moderate- intensity (65–85% HRmax and 12–13 RPE) Placebo Exercise (light-intensity 40% HRmax)	50′	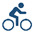 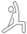	8	QoL, Fatigue, Depression	FACT-G, FACTB, PFS, BDI-II
Dieli (2018) [41]	0–III (CT/RT— post-treatment)	*N* = 91 Exc = 46 UC = 45)	53.5 (10.4)	🗹 AE + RE	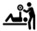	3	Moderate-vigorous (65–85% HRmax) 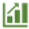	50′ (AE)	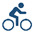 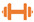	16	QoL, Fatigue, Depression	FACT-B, SF-36, BFI, CES-D
Dong (2019) [42] CEIBISMS	I–III (CT/RT—post-treatment)	*N* = 50 Exc = 26 UC = 24	48.00 (5.54) 51.63 (7.49)	🗹 (Internet and social media app) AE + RE	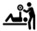	4 (AE) 3 (RE)	not reported (RPE)	30′	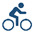 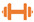	12	QoL	SF-36
Ergun (2013) [43]	I–III A (CT/RT/mastectomy, axillary dissection and sentinel lymph node biopsy—post-treatment)	*N* = 60 Exp (1) = 20 Exp (2) = 20 UC = 20	49.65 (8.25) 55.05 (6.85) 55.30 (10.37)	🗹 AE + RE + Brisk walk 🗵 Brisk walk	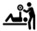 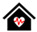	3 + 3 3	Moderate	45′ + 30′ 30′	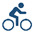 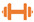 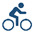	12	QoL, Fatigue, Depression	EORTC QLQ-C30, BFI, BDI
Fernández-Lao (2013) [44]	I–III a (CT/RT/HT post-CT treatment)	*N* = 98 Exc L = 31 Exc W = 33 UC = 34	49 (8) 48 (7) 48 (8)	Multimodal (AE + RE) 🗹 Exp L = Land 🗹 Exp W = Water	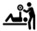	3	60% HRmax	60′	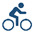 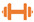	8	QoL, Body image	EORTC QLQ-BR23
Fillion (2008) [45]	0–III (RT + CT/HT—post-treatment)	*N* = 87 Exc = 44 UC = 43	53.09 (9.65) 51.84 (10.25)	🗹 AE + Psycho-educative and fatigue management sessions	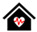	3–5	65–75% HRmax	20–30′	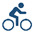	10	Fatigue, Energy level, QoL, Emotional distress	MFI, Vigor-POMS, SF-12, POMS anxiety + depression
Galiano-Castillo (2016) [46] e-CUIDATE	0–III (CT/RT—adjuvant therapy/except HT)	*N* = 81 Exc = 40 UC = 41	47.4 (9.6) 49.2 (7.9)	🗹 AE + RE Internet-based	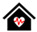	3	Moderate	90′	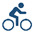 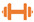	8	QoL, Fatigue, Body image	EORTC QLQ-C30, R-PFS
Ghavami and Akyolcu (2017) [47]	I–III (CT/RT— Post-treatment)	*N* = 80 Exc (*N* = 40) UC = 40	48.75 (9.49) 49.23 (9.46)	🗹 AE Life-style intervention (dietary energy-restriction)	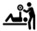	3–5	70–85% HRR	45–60′	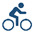	24	Fatigue, Quality of sleep, QoL	CFS, PSQI, EORTC QLQ-C30, QLQ-BR23
Gokal (2016) [48]	I–III (CT—during CT treatment)	*N* = 50 Exc = 25 UC = 25	52 (11.7) 52 (8.9)	🗹 AE	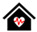	5	Moderate (brisk walking)	10–30′	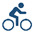	12	Anxiety, Depression, Fatigue, Self-esteem, Mood	HADS, FACT-F Rosenberg S-ES, POMS-SF
Hagstrom (2016) [49]	I–III a (CT/RT/HT—post- CT/ treatment)	*N* = 39 Exc = 20 UC =19	51.2 (8.5) 52.7 (9.4)	🗹 RE	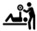	3	8RM (VPA) (3 sets of 8–10 reps of 6 full body exercises)	60′	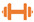	16	QoL, Fatigue	FACIT-F, FACT-G
Harvie (2019) [50]	Early stage (CT/RT/HT —during CT treatment	*N* = 409 Exc C = 137 Exc H =134 UC =138	54.0 (9.2) 54.6 (11.2) 55.3 (10.5)	🗹 Exp C = Community Exp H = Home (phone and mail programme)	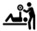 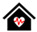	5 (AE) 2 (RE)	Moderate (50–80% HRmax)	30′ (AE) + 10′ (RE)	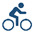 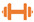	52	Fatigue	FACT-TOI
Hayes (2013) [51] Exercise for Health	0–III (CT/RT/HT—Pre, during and/or post- intervention)	*N* = 194 Exc FtF = 67 Exc Tel = 67 UC = 60	51.2 (8.8) 52.2 (8.6) 53.9 (7.7)	🗹 Exp FtF = (Face-to-face) AE + RE 🗹 Exp Tel = (Telephone) AE + RE	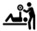 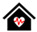	≥4 (AE) ≥2 (RE)	Low-moderate to moderate-high 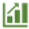	≥45′	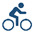 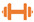	24	QoL, Fatigue, Anxiety, Depression	FACT-B + 4, FACIT-F
Herrero (2006) [52]	I–II of ductal brest carcinoma (CT/RT— Post-treatment)	*N* = 16 Exc = 8 UC = 8	50 (5) 51(10)	🗹 AE + RE	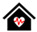	3	70–80% HRmax (AE) 12–15 to 8–10RM (RE) 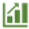	90′	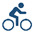 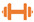	8	QoL	EORTC QLQ-C30,
Huang (2019) [53]	I-III (CT/ RT/ HT – scheduled for adjuvant CT	N= 159 Exc= 81 UC= 78	48.32 (7.90) 48.31 (8.65)	🗹 AE	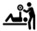	3–5	30–70% HRR	12–25′ to 35–40′	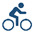	12	Anxiety and Depression	BFI, HADS-A, HADS-D, PSQI
Hwang (2008) [54]	Early stage (RT—waiting list for RT)	*N* = 40 Exc = 17 UC = 23	46.3 (7.5) 46.3 (9.5)	🗹 AE + RE Con = self-shoulder stretching exercise	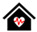	3	50–70% HRmax	50′	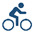 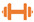	5	QoL, Fatigue	WHOQOL-BREF, BFI
Ligibel (2016) [55]	Metastatic BC (endocrine therapy vs. CT/biologic therapy—during treatment)	*N* = 98 Exc = 47 UC = 51	49.3 (9.6) 50.7 (9.4)	🗹 AE	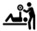 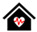	Not reported	Moderate	150′ /wk	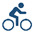	16	QOL, Fatigue	EORTC QLQ C-30, HADS, FACIT-F
Mijwel (2019) [56] OptiTrain	I–III a (CT—during adjuvant CT treatment)	*N* = 206 Exc AE = 72 Exc RE = 74 UC =60	54.4 (10.3) 52.7 (10.3) 52.6 (10.2)	🗵 AE-HIIT 🗵 RE-HIIT	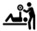	2	Moderate (AE) High, 70–80% 1RM (RE)	60′	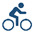 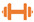	16	Fatigue, QoL, Distress	PFS, EORTC-QLQ-C30, MSAS
Milne (2008) [57]	Early stage (CT/RT—Post-treatment w or w/o HT	*N* = 58 Exc = 29 UC = 29	55.2 (8.4) 55.1 (8.0)	🗵 AE + RE + stretching	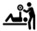	3	(about) 75% HRmax	30′	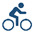 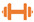 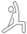	12	QoL, Fatigue, Anxiety	FACT-B, SCFS, SPAS-7
Murtezani (2014) [58]	Early stage (CT/RT—Post-treatment w or w/o HT)	*N* = 62 Exp = 30 UC = 32	53 (11) 51 (11)	🗵 AE	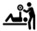	3	50–75% HRR	25–45′	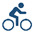	10	QoL	FACT‑B, FACT-G
Musanti (2012) [59]	I–III b (CT/RT— Post-treatment w or w/o HT)	*N* = 42 Exp AE = 10 Exp RE = 9 Exp AR = 11 Con F = 12	51 (5.5) 52 (8.9) 48 (6.7) 52 (7.9)	🗵 AE 🗹 RE 🗹 AE + RE	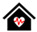	3 3 4–5 (AE) 2 (RE)	40–65% to 85% HRmax 3–5 to 7–8 RPE (0–10 scale)	15–30′	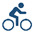 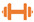 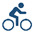 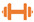	12	Fatigue, Anxiety, Depression, Self-esteem	PFS, HADS, Rosenberg S-ES
Mutrie (2007) [60]	0–III (CT/RT/combined—during and/or post-treatment)	*N* = 174 Exp = 82 UC = 92	51.3 (10.3) 51.8 (8.7)	🗵 AE + RE	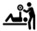	2	50–75% HRmax	45′	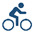 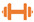	12	QoL, Depression	FACT-G, FACT-B, FACT-F, PANAS, SPAQ, BDI
Naraphong (2015) [61]	I–III a (during CT treatment)	*N* = 23 Exp = 11 UC = 12	46.36 (9.37) 47.17 (6.87)	🗹 AE	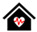	3–5	Low-moderate (40–60% HRmax) weekly increase of 5% of average past week steps 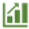	30–40′	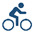	10	Fatigue, Sleep disturbance, Symptom distress	PFS-R, GSD, POMS-BF, MSAS
Ohira (2006) [62] WTBS	I–III, DCIS (CT/RT/HT	*N* = 79 Exp = 39 UC = 40	53.3 (8.7) 52.8 (7.6)	🗹 RE	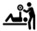	2	10 max/reps	60′	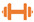	24	QoL, Depression	CARES-SF, CES-D
Paulo (2019) [63]	I–III (CT/RT—undergoing aromatase inhibitor therapy)	*N* = 36 Exp = 18 UC = 18	63.2 (7.1) 66.6 (9.6)	🗹 AE + RE UC = Stretching	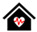 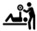	3 2	60–85% HRmax (AE) 15 reps or 65% of max. reps to 8 reps or 80% of max. reps (RE)	30′ (AE) 40′ (RE) 45′	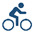 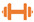 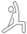	36′	QoL	SF36, EORTC QLQ-C30, EORTC QLQ-BR23
Pinto (2005) [64]	0–II (CT/RT—post CT treatment)	*N* = 82 Exp = 39 UC = 43	53.42 (9.08) 52.86 (10.38)	🗹 AE	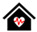	2–5	55–65% HRmax 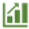	10–30′	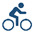	12	Anxiety and depression, Fatigue	POMS, LASF
Reis (2018) [65]	I–IV (CT/RT—during CT/RT treatment)	*N* = 28 Exp =14 UC =14	47.64 (7.60) 45.79 (8.1)	🗹 AE + RE + Flexibility	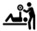 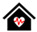	3 (AE + RE) 2 (Flex)	50–60%/80–90% target HR (AE) 3 sets of 12 reps of 12 maximum repetition (RE)	30′ (AE) 30′ (RE) 15′	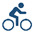 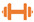 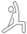	12	Fatigue	PFS-R
Rogers (2015) [66]	I–II BC (CT/RT/HT- DCIS)	*N* = 44 Exp = 20 UC = 24	57.2 ± 5.5 (45–69) 55.2 ± 9.1 (32–67)	🗹 AE + RE	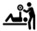 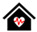	4 (AE) 2 (RE)	Moderate (48–52% HRR)	9–40′	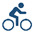 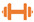	10	Fatigue, Sleep dysfunction, Anxiety, Depression	FSI, fatigue interference, PROMIS
Rogers (2017) [67] BEAT Cancer	I–III A (CT/RT/HT-DCIS)	*N* = 222 Exp = 110 UC = 112	54.4 (8.5)	🗹 AE	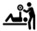 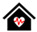	≥3	Moderate (40–59% HRR) 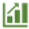	≥60′	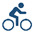	10	Fatigue, Anxiety, Depression	FSI, HAD
Saarto (2012) [68]	Early stage (CT/RT)	*N* = 500 Exp = 263 UC = 237	52.3 (36–68) 52.4 (35–68)	🗹 AE	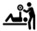 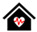	1 2–3	5–7 METs	60′	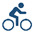	48	QoL, Fatigue, Depression, Body image	EORTC QLQ-C30, BR-23, FACIT-F, RBDI, WHQ
Saxton (2014) [69]	Early stage (post- treatment)	*N* = 85 Exp (*N* = 44) UC =41	55.8 (10.0) 55.3 (8.8)	🗹 AE + RE (+ education about hypocaloric diet intake)	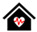	3	65–85% HRmax	40–45′	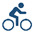 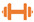	24	Depression, Stress	BDI-II, PSS
Schmidt (2012) [70]	I–III (CT/RT—Post-treatment)	*N* = 33 Exp =15 UC =18	58 (8.41) 55 (10.59)	🗵 RE	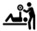	1	50% h 1RM	60′	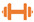	26	QoL	EORTC QLQ C30, BR23
Schmidt (2015) [71] BEATE	Tumor stage 1–4 (CH, HT etc.—during CT treatment	*N* = 95 Exp = 49 UC = 46	52.2 (9.9) 53.3 (10.2)	🗵 RE	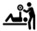	2	60–80% 1RM	60′	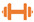	12	Fatigue, QoL, Depression	FAQ, EORTC QLQ C30 + BR23, CES-D
Schmidt (2015b) [72]	Primary moderate- or high-risk BC (CT—during CT treatment)	*N* = 67 Exp AE = 20 Exp RE = 21 UC = 26	56 (10.15) 53 (12.55) 54 (11.19)	🗵AE 🗹 RE	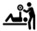	2	11–14 Borg scale (AE) 50% h 1RM (RE)	60′	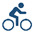 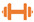	12	QoL Fatigue, Body image	EORTC QLQ C30 + BR23, MFI20
Schmidt (2016) [73] BEST	0–III (RT—During RT)	*N* = 103 Exc = 54 UC = 49	57.1 (8.9) 57.3 (8.8)	🗹 RE	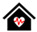	2	3 sets of 8–2 reps of 8 machine-based exercises	60′	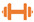	12	Fatigue, QoL	FAQ, QLQ-C30, CES-D
Scott (2013) [74]	I–III (CT/RT/HT—Post-treatment)	*N* = 90 Exc = 47 UC = 43	55.6 (10.2) 55.9 (8.9)	Lifestyle intervention 🗵 AE + RE + hypocaloric healthy eating program	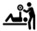	3	65–85% HRmax	30′ (AE) 10–15′ (RE)	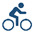 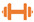	24	QoL	FACT-G, FACT-B
Segal (2001) [75]	I–II (CH/RT HT—during treatment)	*N* = 123 Exc S = 42 Exc SD = 40 UC = 41	51.0 (8.7) 51.4 (8.7) 50.3 (8.7)	🗹 AE (S) Supervised 🗹 AE (SD) Self-directed	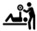 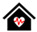	5 3	50–60% VO_2_ max	60′	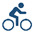 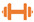	26	QoL	MOS SF-36, FACT-G, FACT-B
Shobeiri (2016) [76]	I–II (CT/RT—Post-treatment w or w/o HT)	*N* = 60 Exc = 30 UC = 30	42.70 (9.6) 43.50 (8.60)	🗵 AE	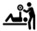	2	50–75% HRR	25–45′	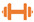	10	Fatigue, QoL, Body image	EORTC QLQ-C30, EORTC QLQ-BR23
Speack (2010) [77] PAL	0–III, DCIS (CT/RT— Post-treatment) W or w/o lymphedema	*N* = 234 Exc = 59 Exc wL = 54 UC = 63 UC wL = 58	55 (7) 58 (9) 57 (8) 58 (9)	🗹 RE 🗹 RE wL * wL = with lymphedema	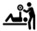	2	not reported	90′	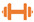 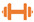	52	Body image, QoL	BIRS, SF-36
Steindorf (2014) [78] BEST	0–III (CT, RT, HT, trastuzumab therapy—during RT)	*N* = 155 Exc = 77 UC = 78	55.2 (9.5) 56.4 (8.7)	🗹 RE	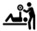	2	60–80% 1RM	60′	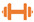	12	Fatigue, QoL, Depression, Body image	FAQ, EORTC QLQ-C30, EORTC QLQ-C23, CES-D,
Travier (2015) [79] PACT	M0 (CT—During; RT after intervention)	*N* =164 Exc = 87 UC = 77	49.7 (8.2) 49.5 (7.9)	🗹 AE + RE	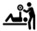	2	Moderate- vigorous 65–75% to 45% 1RM (RE) HR ventilatory threshold (AE)	60′	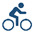	18	Fatigue, QoL, Anxiety, Depression	MFI, FQL, SF-36, QLQ-C30, HADS
Wang (2011) [80]	I–II (CT/RT—before CT treatment)	*N* = 72 Exc = 35 UC = 37	48.40 (10.15) 52.3 (8.84)	🗹 AE	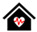	3–5	40–60% HRmax	≥30′	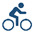	6	QoL, Fatigue, Sleep disturbances,	FACT-G, FACIT-F, PSQI
Winters (2012) [81] POWIR	I–III a (CT/RT—Post-treatment)	*N* = 106 Exc = 52 UC = 54	62.3 (6.7) 62.6 (6.7)	🗹 RE + impact training (POWIR) UC = Stretching placebo program. (Flexibility)	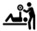	2	60–80% 1RM	60′	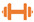 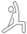	52	Fatigue	SCF, SF-36

Frequency, Intensity, Time and Type (FITT); Duration (D); Patient Reported Outcome (PRO); Exercise intervention group (Exc); Usual care or control group (UC); Aerobic Exercise training (AE); Resistance Exercise training (RE); WHO guidelines of minimum physical activity/exercise recommended (🗹: accomplished; 🗵 not accomplished); Chemotherapy (CT); Radiotherapy (RT); Hormonal Therapy (HT); Quality of Life (QoL); ratings of perceived exertion (RPE); moderate to vigorous physical activity (MVPA); Heart Rate Reserve (HRR); Ductal Carcinoma in Situ (DCIS); PRO measures: Beck Depression Inventory (BDI); The Brief Fatigue Inventory (BFI); European Organization for Research and Treatment of Cancer Quality of Life Questionnaire (EORTC QLQ-C30); Finnish modified version of Beck’s 13-item depression scale (RBDI); Functional Assessment of Cancer Therapy, FACT-Anemia (FACT-A); Functional Assessment of Cancer Therapy-Anemia (FACT-An); Breast (FACT-B); Fatigue (FACT-F); General (FACT-G); Functional Assessment of Chronic Illness Therapy (FACIT); questionnaire for fatigue (FACIT-F); Medical Outcomes Study Short Form (MOS SF-36); Piper Fatigue Scale (PFS); Schwartz Cancer Fatigue Scale (SCF); Women’s Health Questionnaire (WHQ); Patient Reported Outcomes Measurement Information System (PROMIS); Body Image Scale (BIS); Happiness Measure (HM); Social Physique Anxiety Scale-7 items (SPAS-7); Memorial Symptom Assessment Scale (MSAS); Fatigue Assessment Questionnaire (FAQ); Hospital Anxiety and Depression Scale (HADS); HADS-Anxiety (HADS-A); HADS-Depression (HADS-D); Pittsburgh Sleep Quality Index (PSQI); Rosenberg Self-Esteem scale (S-ES); Center for Epidemiological Studies Depression Scale (CES-D); Spielberger State Anxiety Inventory (SAI); Physical Function scale (SF-36); World Health Organization Quality of Life-BREF (WHOQOL-BREF); State-Trait Anxiety Inventory, (STAI); STAI-State Anxiety (STAI-YI), Cohen’s 10-item Perceived Stress Scale (10-PSS); Trial Outcome Index-Anemia (TOI-An) subscale and fatigue subscale; Health Related Quality of Life (HRQoL); Profile of Mood States (POMS); Linear analog scale for fatigue (LASF); Cancer Rehabilitation Evaluation System-Short Form (CARES-SF); Body Image and Relationships Scale (BIRS). * Age presented with mean and SD or range where reported. 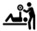 Supervised intervention; 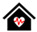 Home-based intervention; 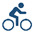 Aerobic exercise; 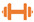 Resistance exercise/strength training; 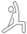 Flexibility/Stretching exercises; 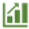 Progressive; 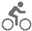 Aerobic exercise (control).

## Data Availability

No new data were created or analyzed in this study. Data sharing is not applicable to this article.

## Supplementary Materials

The following are available online at https://www.mdpi.com/2072-6694/13/2/264/s1. Table S1. Search strategy implemented in three electronic databases and results of total records Table S2. Assessment of methodological quality and risk of bias with PEDro scale, Table S3. Exercise effects on mental health parameters, Table S4. Exercise effects on mental health parameters according to training programs characteristics, Figure S5. Forest plot about the exercise effects on anxiety, Figure S6. Forest plot about the exercise effects on body image, Figure S7. Forest plot about the exercise effects on depression, Figure S8. Forest plot about the exercise effects on fatigue, Figure S9. Forest plot about the exercise effects on happiness, Figure S10. Forest plot about the exercise effects on self-esteem, Figure S11. Forest plot about the exercise effects on sleep disturbance, Figure S12. Forest plot about the exercise effects on stress, Figure S13. Forest plot about the exercise effects on overall quality of life, Figure S14. Forest plot about the exercise effects on FACT-B questionnaire, Figure S15. Forest plot about the exercise effects on emotional function, Figure S16. Association between effects of exercise interventions on anxiety and its duration, Figure S17. Association between effects of exercise interventions on body image and its duration, Figure S18. Association between effects of exercise interventions on depression and its duration, Figure S19. Association between effects of exercise interventions on fatigue and its duration, Figure S20. Association between effects of exercise interventions on happiness and its duration, Figure S21. Association between effects of exercise interventions on self-esteem and its duration, Figure S22. Association between effects of exercise interventions on sleep disturbance and its duration, Figure S23. Association between effects of exercise interventions on stress and its duration, Figure S24. Association between effects of exercise interventions on overall quality of life and its duration, Figure S25. Association between effects of exercise interventions on FACT-B questionnaire and its duration, Figure S26. Association between effects of exercise interventions on emotional function and its duration, Figure S27. Doi plot depicting possible small-study effects on anxiety, Figure S28. Doi plot depicting possible small-study effects on body image, Figure S29. Doi plot depicting possible small-study effects on depression, Figure S30. Doi plot depicting possible small-study effects on fatigue, Figure S31. Doi plot depicting possible small-study effects on happiness, Figure S32. Doi plot depicting possible small-study effects on self-esteem, Figure S33. Doi plot depicting possible small-study effects on sleep disturbance, Figure S34. Doi plot depicting possible small-study effects on stress, Figure S35. Doi plot depicting possible small-study effects on overall quality of life, Figure S36. Doi plot depicting possible small-study effects on FACT-B questionnaire, Figure S37. Doi plot depicting possible small-study effects on emotional function.
