# Supplementary material for: Evidence-Based Exercise Recommendations to Improve Mental Wellbeing in Women with Breast Cancer during Active Treatment: A Systematic Review and Meta-Analysis
Source: Cancers (Basel). 2021 Jan 12;13(2):264. doi: 10.3390/cancers13020264 (PMC7828164; doi:10.3390/cancers13020264)
Supplement: Supplementary file 1 [file cancers-13-00264-s001.pdf]

Supplementary Material: Evidence-Based Exercise Recommendations to Improve Mental Wellbeing in Women with Breast Cancer During Active Treatment: A Systematic Review and Meta-Analysis

Robinson Ramírez-Vélez, Fabiola Zambom-Ferraresi, Antonio García-Hermoso, Justina Kievisiene, Alona Rauckiene-Michealsson and César Agostinis-Sobrinho

Table S1. Search strategy implemented in three electronic databases and results of total records.

Pubmed	((randomized controlled trial[Publication Type]) OR (controlled clinical trial[Publication Type]) OR ("randomi*"[Title/Abstract]) OR (trial[Title]) OR (clinical trials[MeSH Major Topic])) AND (breast[Title/Abstract]) AND ((cancer[Title/Abstract]) OR (neoplasm[Title/Abstract]) OR ("tumour*"[Title/Abstract]) OR ("tumor*"[Title/Abstract]) OR ("carcino*"[Title/Abstract]) OR ("Leukaemi*"[Title/Abstract]) OR ("Leukemi*"[Title/Abstract])) AND ((Physical activity[Title/Abstract]) OR (exercise[Title/Abstract]) OR (aerobic[Title/Abstract]) OR (endurance[Title/Abstract]) OR (resistance[Title/Abstract]) OR (strength[Title/Abstract]) OR (flexibility[Title/Abstract]) OR (stretching[Title/Abstract])) AND ((mental wellbeing[Title/Abstract]) OR (mental health[Title/Abstract]) OR (mood states[Title/Abstract]) OR (anxiety[Title/Abstract]) OR (depression[Title/Abstract]) OR ("quality of life"[Title/Abstract]) OR (self-esteem[Title/Abstract]) OR (fatigue[Title/Abstract]))	651
WOS	TS= (randomized controlled trial OR cross over controlled trial* OR controlled clinical trial OR randomi* OR trial) AND TI=breast AND TI= (cancer OR neoplasm* OR tumour* OR tumor* OR carcino* leukaemi* OR leukemi*) AND TI= (physical activity OR exercise OR aerobic OR endurance OR resistance OR strength OR flexibility OR stretching) AND AB= (mental wellbeing OR mental health OR mood states OR anxiety OR depression OR quality of life OR self-esteem OR fatigue)	443
Ovid	TI (randomized controlled trial OR cross over controlled trial* OR controlled clinical trial OR randomi* OR trial) AND TI breast AND TI (cancer OR neoplasm* OR tumour* OR tumor* OR carcino* leukaemi* OR leukemi*) AND TI (physical activity OR exercise OR aerobic OR endurance OR resistance OR strength OR flexibility OR stretching) AND AB (mental wellbeing OR mental health OR mood states OR anxiety OR depression OR quality of life OR self-esteem OR fatigue)	378
	**Overall**	**1472**

N = 1472, duplicates = 875 (Eliminated with Zotero).

Table S2. Assessment of methodological quality and risk of bias with PEDro scale.

Study	Q1	Q2	Q3	Q4	Q5	Q6	Q7	Q8	Q9	Q10	Total (0 to 10)
Adams (2016) [25]	Y	Y	Y	N	N	N	N	N	Y	Y	5
Ammitzboll (2019) [26]	Y	N	Y	N	N	N	N	Y	Y	Y	5
Baglia (2019) [27]	Y	Y	Y	N	N	N	Y	Y	Y	Y	7
Basen-Engquist (2020) [28]	Y	Y	Y	N	N	N	Y	Y	Y	N	6
Bloomquist (2019) [29]	Y	N	Y	N	N	Y	N	Y	Y	Y	6
Cadmus (2009) [30]	Y	Y	Y	N	N	N	Y	Y	Y	Y	7
Campbell (2005) [31]	Y	N	Y	N	N	N	Y	N	Y	Y	5
Cantarero-Villanueva (2013) [32]	Y	Y	Y	N	N	Y	Y	N	Y	Y	7
Cantarero-Villanueva (2011) [33]	Y	Y	Y	N	N	Y	N	Y	Y	Y	7
Carayol (2019) [34]	Y	Y	Y	N	N	N	Y	Y	Y	Y	7
Casla (2015) [35]	Y	Y	Y	N	N	N	Y	N	Y	Y	6
Cormie (2013) [36]	Y	Y	Y	N	N	N	Y	Y	Y	Y	7
Cornette (2016) [37]	Y	N	Y	N	N	N	N	Y	Y	Y	5
Courneya (2003) [38]	Y	N	Y	N	N	Y	Y	Y	Y	Y	7
Courneya (2007) [39]	Y	Y	Y	N	N	N	Y	Y	Y	Y	7

Daley (2007) [40]	Y	Y	Y	N	N	N	Y	Y	Y	Y	7
Dieli (2018) [41]	Y	Y	Y	N	N	N	Y	N	Y	Y	6
Dong (2019) [42]	Y	Y	Y	N	N	Y	N	N	Y	Y	6
Ergun (2013) [43]	Y	N	Y	N	N	Y	Y	N	Y	Y	6
Fernández-Lao (2013) [44]	Y	Y	Y	N	N	N	Y	Y	Y	Y	7
Fillion (2008) [45]	Y	Y	Y	N	N	N	Y	Y	Y	Y	7
Galiano-Castillo (2016) [46]	Y	Y	Y	N	N	Y	Y	Y	Y	Y	8
Ghavami and Akyolcu (2017) [47]	Y	Y	Y	N	N	N	Y	N	Y	Y	6
Gokal (2016) [48]	Y	N	Y	N	N	N	N	Y	Y	Y	5
Hagstrom (2016) [49]	Y	Y	Y	N	N	N	Y	Y	Y	Y	7
Harvie (2019) [50]	Y	N	Y	N	N	N	Y	Y	Y	Y	6
Hayes (2013) [51]	Y	N	Y	N	N	Y	Y	Y	Y	Y	7
Herrero (2006) [52]	Y	Y	Y	N	N	Y	N	N	Y	Y	6
Huang (2019) [53]	Y	N	Y	N	N	Y	N	Y	Y	Y	6
Hwang (2008) [54]	Y	N	Y	N	N	N	Y	N	Y	Y	5
Ligibel (2016) [55]	Y	N	Y	N	N	Y	Y	N	Y	Y	6
Mijwel (2019) [56]	Y	N	Y	N	N	N	Y	Y	Y	Y	6
Milne (2008) [57]	Y	Y	Y	N	N	N	Y	Y	Y	Y	7
Murtezani (2014) [58]	Y	N	Y	N	N	Y	N	N	Y	Y	5
Musanti (2012) [59]	Y	Y	Y	N	N	Y	N	N	Y	Y	6
Mutrie (2007) [60]	Y	Y	Y	N	N	Y	Y	Y	Y	Y	8
Naraphong (2015) [61]	Y	Y	Y	N	N	N	Y	Y	Y	Y	7
Ohira (2006) [62]	Y	N	Y	N	N	Y	Y	N	Y	Y	6
Paulo (2019) [63]	Y	N	Y	N	N	N	N	Y	Y	Y	5
Pinto (2005) [64]	Y	N	T	N	N	N	Y	Y	Y	Y	6
Reis (2018) [65]	Y	Y	Y	N	N	Y	Y	Y	Y	Y	8
Rogers (2015) [66]	Y	Y	N	N	N	Y	Y	Y	Y	Y	7
Rogers (2017) [67]	Y	Y	N	N	N	Y	Y	Y	Y	Y	7
Saarto (2012) [68]	Y	N	Y	N	N	N	Y	N	Y	Y	5
Saxton (2104) [69]	Y	Y	Y	N	N	Y	Y	Y	Y	Y	8
Schmidt (2012) [70]	Y	N	Y	N	N	N	Y	N	Y	Y	5
Schmidt (2015) [71]	Y	Y	Y	N	N	N	Y	Y	Y	Y	7
Schmidt (2015b) [72]	Y	Y	Y	N	N	N	N	N	Y	Y	5
Schmidt (2016) [73]	Y	N	Y	N	N	N	N	Y	Y	Y	5
Scott (2013) [74]	Y	Y	Y	N	N	Y	Y	Y	Y	Y	8
Segal (2001) [75]	Y	N	Y	N	N	N	N	Y	Y	Y	5
Shobeiri (2016) [76]	Y	N	Y	N	N	N	Y	Y	Y	Y	6
Speck (2010) [77]	Y	N	Y	N	N	Y	N	N	Y	Y	5
Steindorf (2014) [78]	Y	Y	Y	N	N	N	Y	Y	Y	Y	7
Travier (2015) [79]	Y	Y	Y	N	N	Y	Y	Y	Y	Y	8
Wang (2011) [80]	Y	N	Y	N	N	N	Y	N	Y	Y	5
Winters (2012) [81]	Y	Y	Y	N	N	Y	N	Y	Y	Y	7

Q1: Random allocation; Q2: Concealed allocation; Q3: Groups similar at baseline; Q4: Participant blinding; Q5: Therapist Blinding; Q6: Assessor Blinding; Q7: Adequate follow-up; Q8: Intention to treat analysis; Q9: Between-group difference reported; Q10: Point estimate and variability reported.

Table S3. Exercise effects on mental health parameters.

**Mental health parameter**	**Studies (*n*)**	**Cohen's d**	**95% CI**	***p***	***I*²**	***Egger***
Anxiety	10	−0.22	−0.43 to −0.01	0.039	53.0	0.988
Body image	12	0.27	0.01 to 0.54	0.046	69.2	0.542
Depression	17	−0.24	−0.40 to −0.07	0.008	66.6	0.210
Fatigue	39	−0.47	−0.60 to −0.34	<0.001	69.8	<0.001
Happiness	3	0.29	−0.46 to 1.03	0.280	21.5	0.651
Self-esteem	10	0.24	−0.01 to 0.50	0.081	41.5	0.731

Sleep disturbance	4	−0.68	−2.12 to 0.76	0.229	88.1	0.354
Stress	4	−0.24	−0.66 to 0.18	0.843	0	0.394
Health-Related Quality of Life						
Overall	20	0.46	0.24 to 0.68	<0.001	71.6	<0.001
FACT-B	13	0.60	0.27 to 0.93	<0.001	76.2	0.267
Emotional function	21	0.33	0.16 to 0.50	<0.001	65.7	0.002

Table S4. Exercise effects on mental health parameters according to training programs characteristics.

Mental health parameter	Studies (n)	Cohen´s d	95% CI	p	I^2
**Fatigue**					
Aerobic training	16	−0.49	−0.75 to −0.24	0.001	78.9
Resistance training	9	−0.23	−0.43 to −0.03	0.029	27.2
Concurrent training	16	−0.59	−0.79 to −0.38	<0.001	62.5
<150 min/week	24	−0.37	−0.51 to −0.23	<0.001	59.3
≥150 min/week	13	−0.65	−0.93 to −0.38	<0.001	76.3
Moderate intensity	21	−0.50	−0.66 to −0.34	<0.001	48.0
Moderate-to-vigorous intensity	15	−0.28	−0.47 to −0.09	0.008	64.7
Vigorous intensity	5	−0.97	−1.70 to −0.24	0.021	71.7
**Stress**					
Aerobic training	3	−0.20	−0.91 to 0.50	0.339	0
**Self-esteem**					
Aerobic training	7	0.28	−0.08 to 0.64	0.107	53.7
Resistance training	3	0.18	−0.60 to 0.97	0.417	17.3
<150 min/week	7	0.22	−0.15 to 0.60	0.199	50.0
≥150 min/week	3	0.28	−0.51 to 1.07	0.268	38.2
Moderate intensity	5	0.15	−0.34 to 0.63	0.450	51.7
Moderate-to-vigorous intensity	3	0.28	−0.51 to 1.07	0.268	38.2
**Anxiety**					
Aerobic training	5	−0.15	−0.53 to 0.23	0.333	54.3
Concurrent training	4	−0.35	−0.97 to 0.27	0.172	69.7
<150 min/week	6	−0.23	−0.55 to 0.09	0.126	59.5
≥150 min/week	4	−0.21	−0.73 to 0.31	0.290	52.2
Moderate intensity	5	−0.47	−0.72 to −0.22	0.006	0
Moderate-to-vigorous intensity	5	−0.02	−0.25 to 0.21	0.827	0
**Depression**					
Aerobic training	7	−0.20	−0.49 to 0.08	0.131	65.1
Resistance training	3	−0.03	−0.46 to 0.40	0.809	0
Concurrent training	7	−0.37	−0.72 to −0.02	0.043	70.7
<150 min/week	11	−0.16	−0.36 to 0.03	0.088	64.0
≥150 min/week	6	−0.39	−0.75 to −0.04	0.037	50.7
Moderate intensity	4	−0.28	−0.66 to 0.10	0.102	31.9
Moderate-to-vigorous intensity	13	−0.24	−0.44 to −0.07	0.028	70.9
**Body image**					
Aerobic training	3	0.70	−0.57 to 1.98	0.141	71.3
Resistance training	4	0.12	−0.61 to 0.85	0.631	79.8
Concurrent training	5	0.12	−0.17 to 0.41	0.309	0
<150 min/week	6	0.36	−0.03 to 0.75	0.062	62.5
≥150 min/week	6	0.18	−0.35 to 0.71	0.432	75.2
Moderate intensity	6	0.02	−0.25 to 0.30	0.840	0
Moderate-to-vigorous intensity	3	0.42	−0.78 to 1.61	0.272	72.7
**Sleep disturbance**					
Aerobic training	3	−0.83	−3.42 to 1.77	0.303	89.9
**Health-Related Quality of Life Overall**					
Aerobic training	10	0.58	0.15 to 1.01	0.014	83.8

Resistance training	6	0.24	−0.01 to 0.48	0.056	0
Concurrent training	4	0.55	−0.55 to 1.65	0.209	69.4
<150 min/week	15	0.49	0.25 to 0.73	0.001	62.6
≥150 min/week	5	0.40	−0.42 to 1.22	0.250	75.8
Moderate intensity	7	0.48	0.20 to 0.76	0.006	0
Moderate-to-vigorous intensity	8	0.38	−0.13 to 0.89	0.119	80.8
Vigorous intensity	3	0.48	−0.34 to 1.31	0.495	0
**Emotional function**					
Aerobic training	9	0.36	0.04 to 0.68	0.032	70.1
Resistance training	6	0.20	−0.12 to 0.52	0.174	43.6
Concurrent training	6	0.43	−0.03 to 0.89	0.063	72.5
<150 min/week	13	0.30	0.03 to 0.57	0.033	73.1
≥150 min/week	8	0.39	0.21 to 0.58	0.001	0
Moderate intensity	8	0.34	0.08 to 0.61	0.017	41.7
Moderate-to-vigorous intensity	9	0.25	−0.09 to 0.60	0.128	77.0
Vigorous intensity	4	0.58	0.07 to 1.09	0.036	0
**FACT-B**					
Aerobic training	6	0.43	−0.01 to 0.86	0.051	59.7
Concurrent training	7	0.76	0.18 to 1.35	0.019	83.0
<150 min/week	6	0.83	0.14 to 1.53	0.027	82.7
≥150 min/week	7	0.45	0.06 to 0.84	0.029	68.6
Moderate intensity	5	0.82	−0.03 to 1.66	0.055	82.4
Moderate-to-vigorous intensity	7	0.43	0.01 to 0.86	0.048	73.5

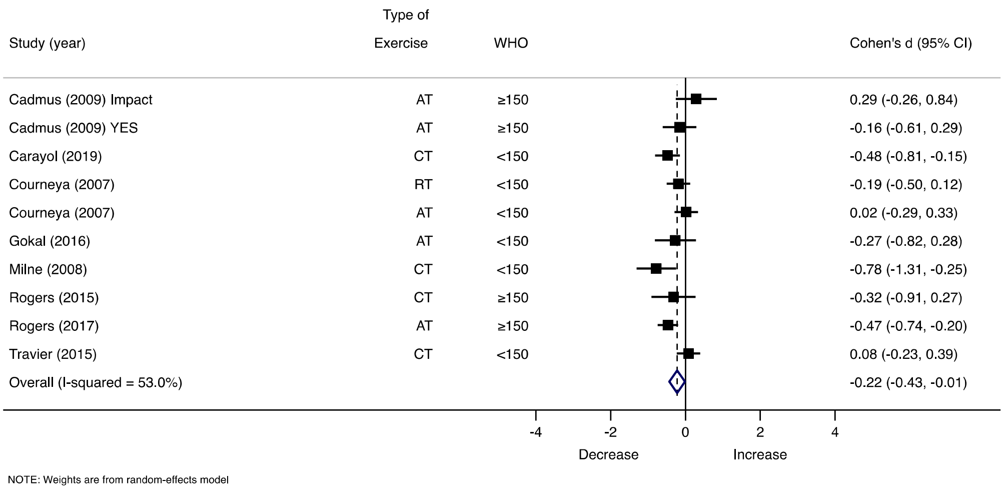

Figure S5. Forest plot about the exercise effects on anxiety. AT, aerobic training; CT, concurrent training.

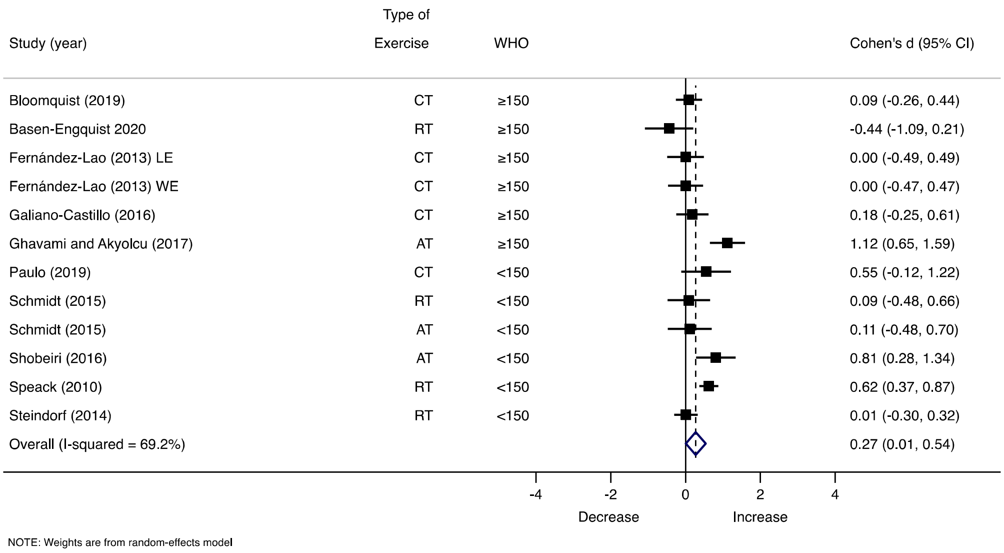

Figure S6. Forest plot about the exercise effects on body image. AT, aerobic training; CT, concurrent training; RT, resistance training.

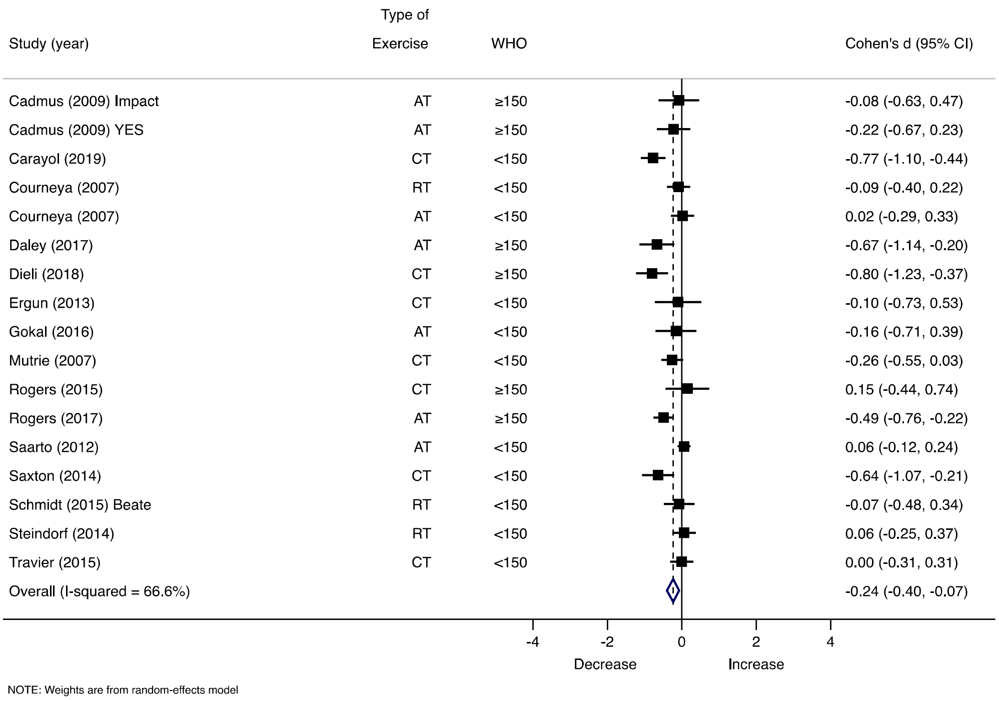

Figure S7. Forest plot about the exercise effects on depression. AT, aerobic training; CT, concurrent training; RT, resistance training.

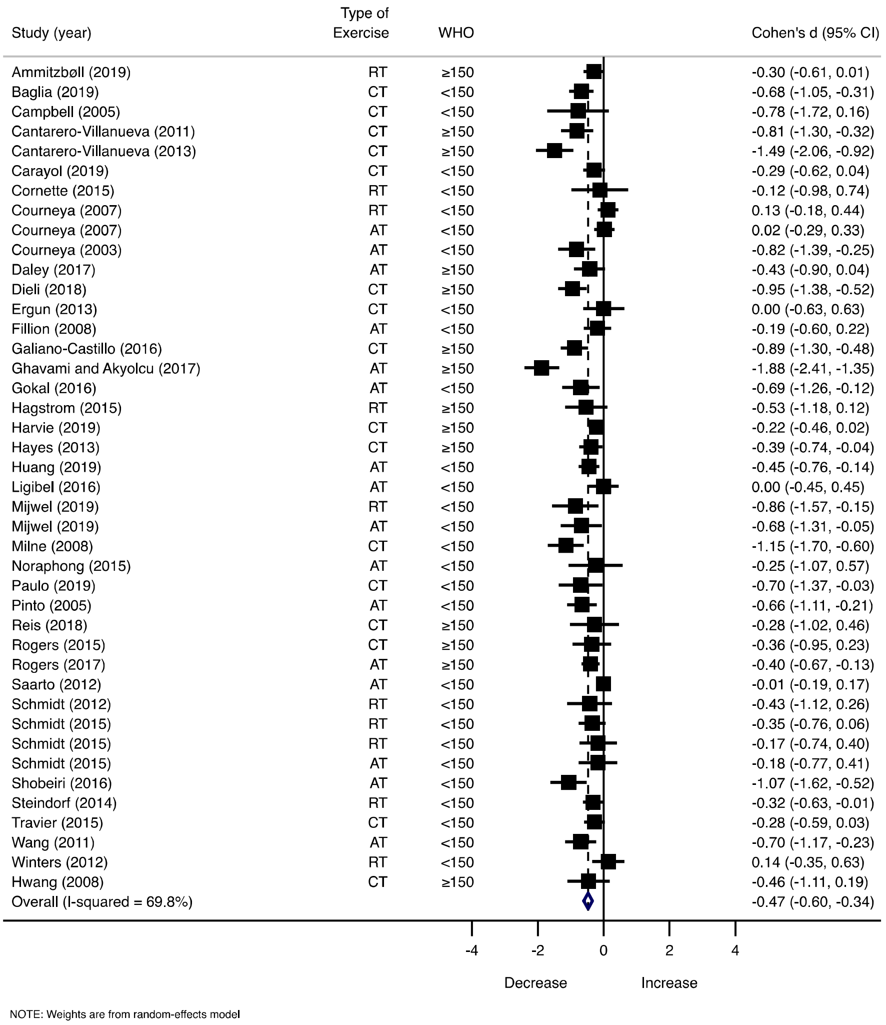

Figure S8. Forest plot about the exercise effects on fatigue. AT, aerobic training; CT, concurrent training; RT, resistance training.

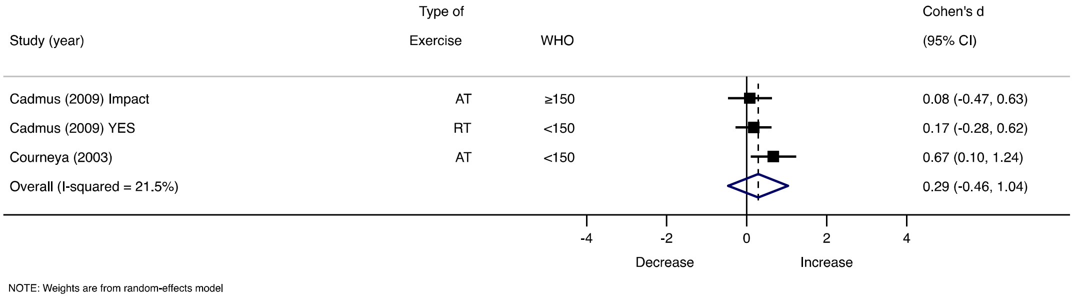

Figure S9. Forest plot about the exercise effects on happiness. AT, aerobic training; RT, resistance training.

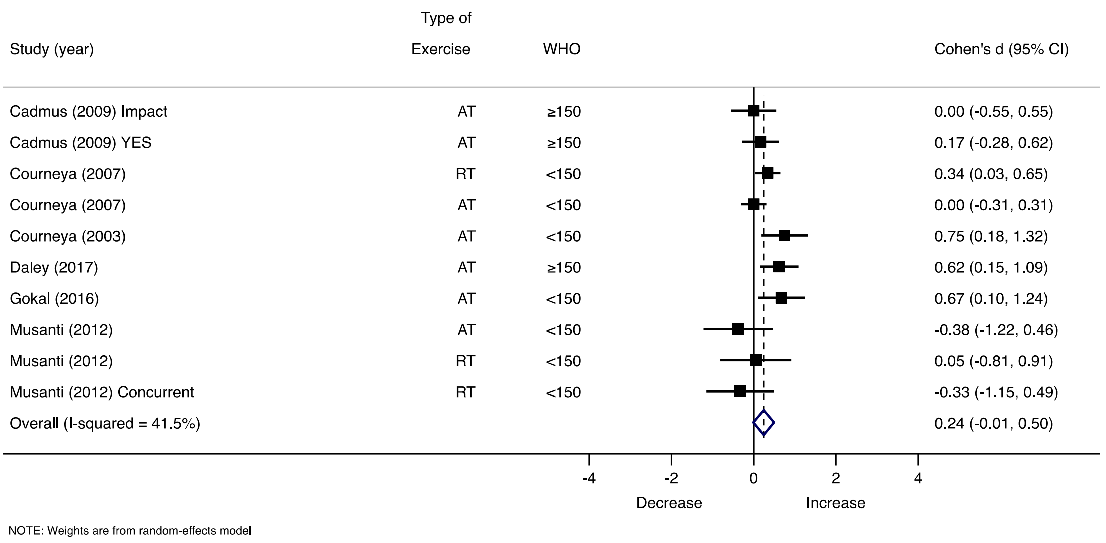

Figure S10. Forest plot about the exercise effects on self-esteem. AT, aerobic training; RT, resistance training.

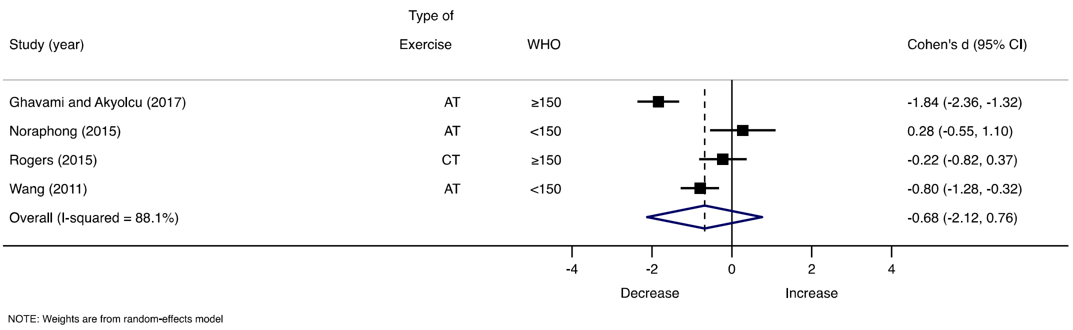

Figure S11. Forest plot about the exercise effects on sleep disturbance. AT, aerobic training; CT, concurrent training.

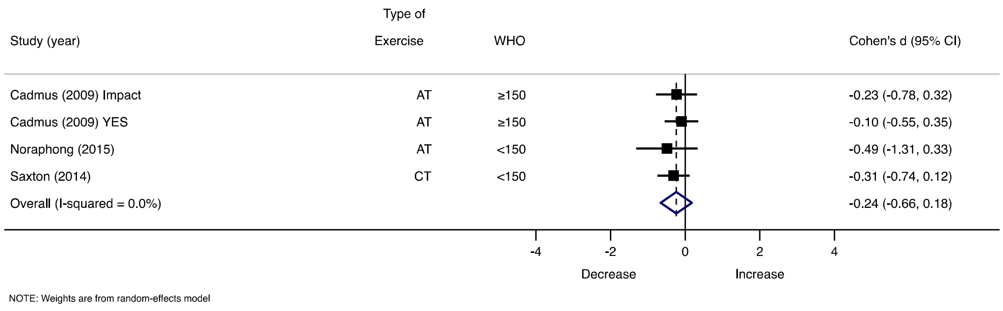

Figure S12. Forest plot about the exercise effects on stress. AT, aerobic training; CT, concurrent training.

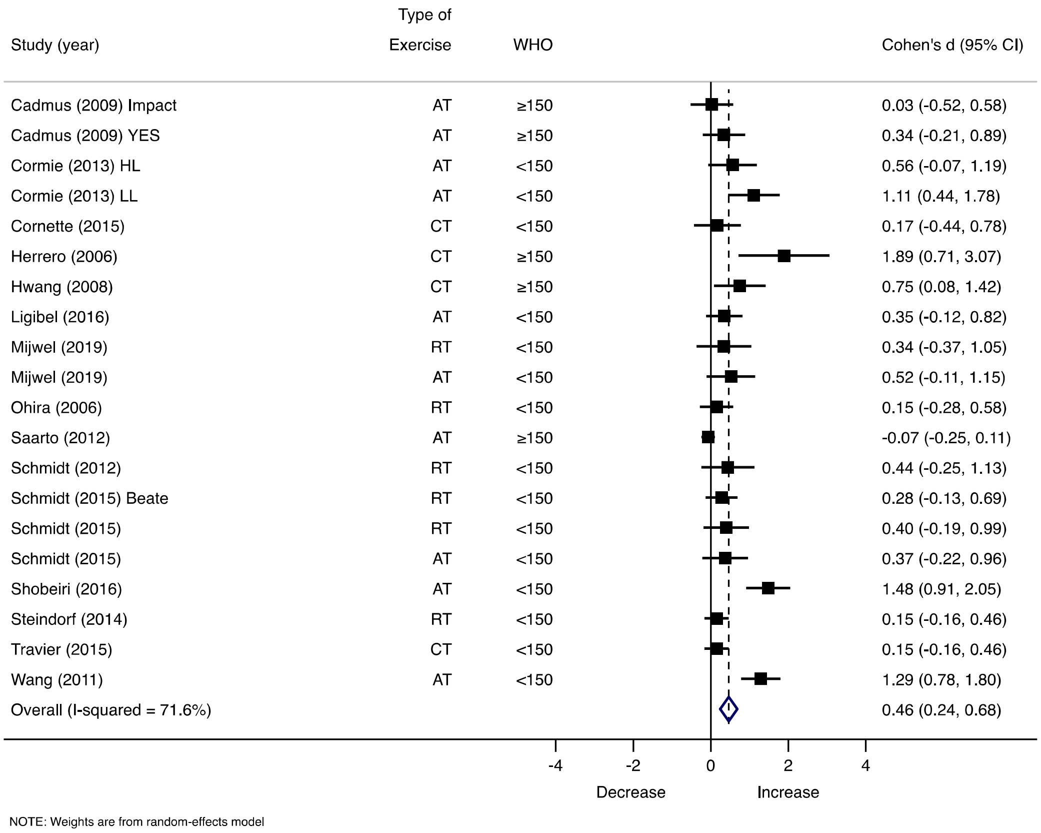

Figure S13. Forest plot about the exercise effects on overall quality of life. AT, aerobic training; CT, concurrent training; RT, resistance training.

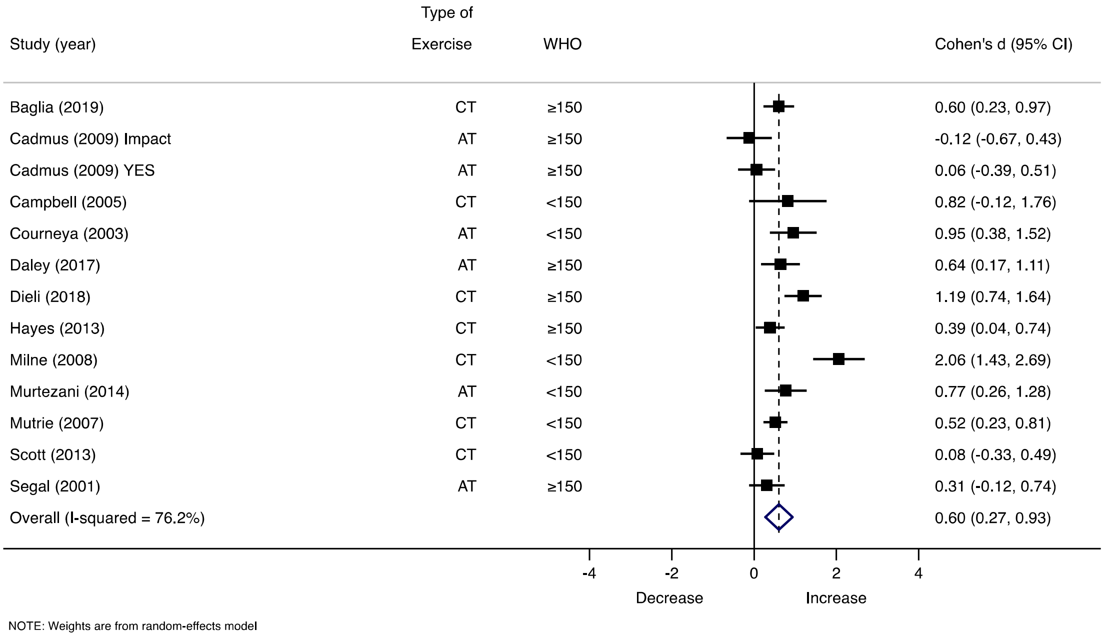

Figure S14. Forest plot about the exercise effects on FACT-B questionnaire. AT, aerobic training; CT, concurrent training.

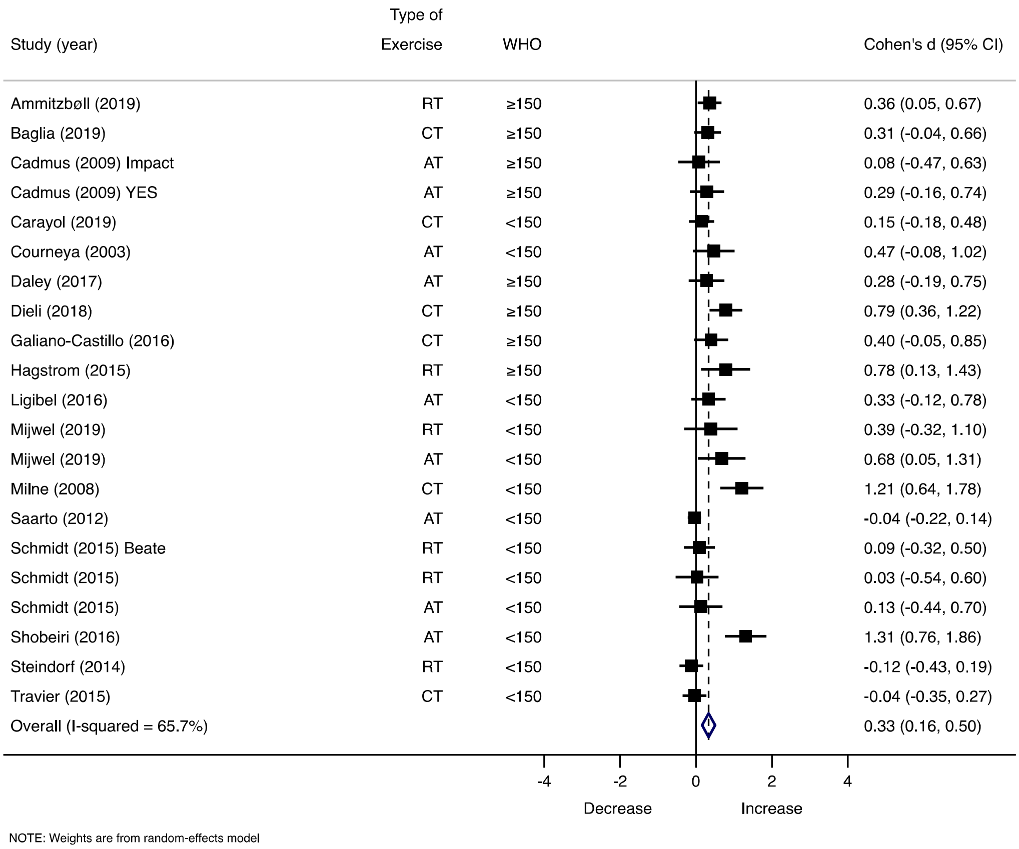

Figure S15. Forest plot about the exercise effects on emotional function. AT, aerobic training; CT, concurrent training; RT, resistance training.

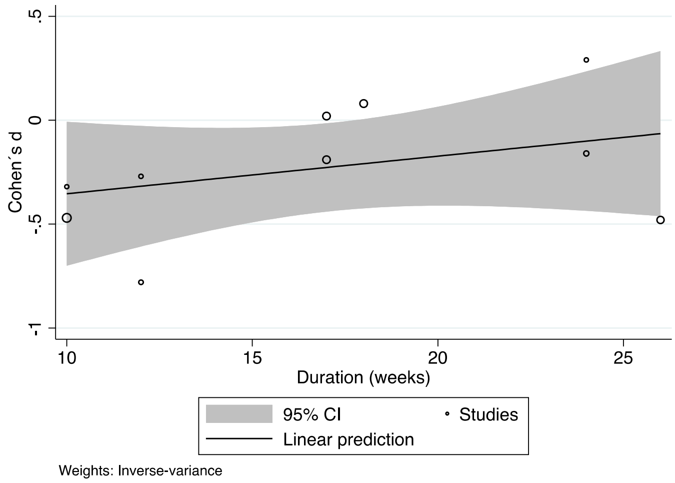

Figure S16 Association between effects of exercise interventions on anxiety and its duration.

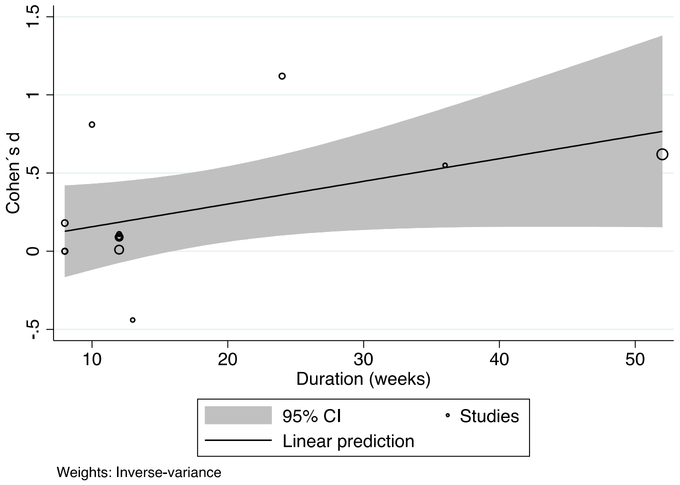

Figure S17 Association between effects of exercise interventions on body image and its duration.

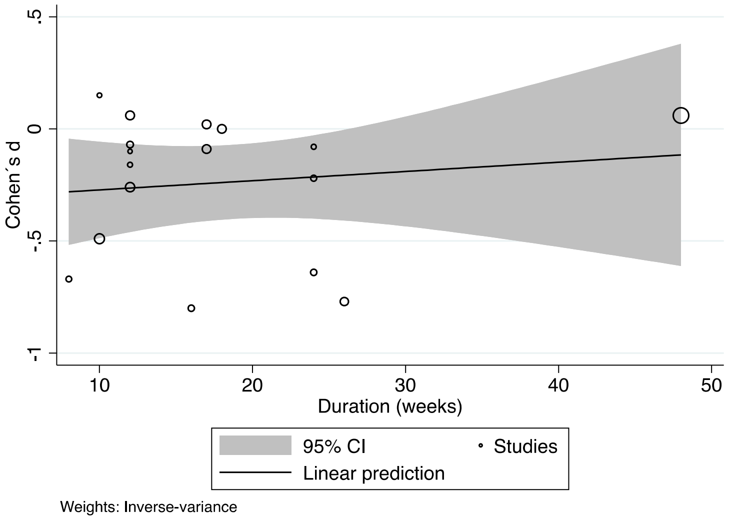

Figure S18 Association between effects of exercise interventions on depression and its duration.

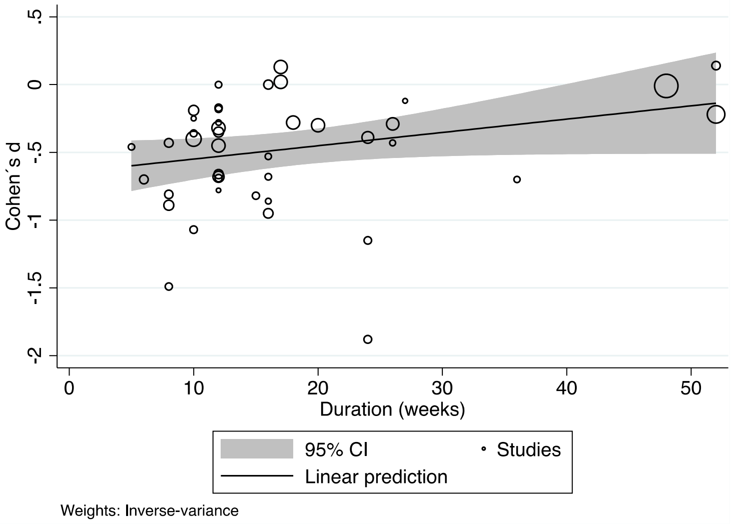

Figure S19 Association between effects of exercise interventions on fatigue and its duration.

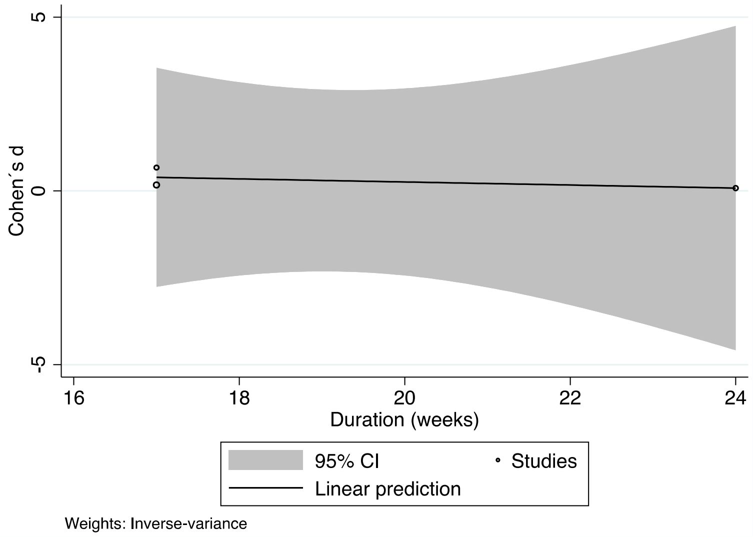

Figure S20 Association between effects of exercise interventions on happiness and its duration.

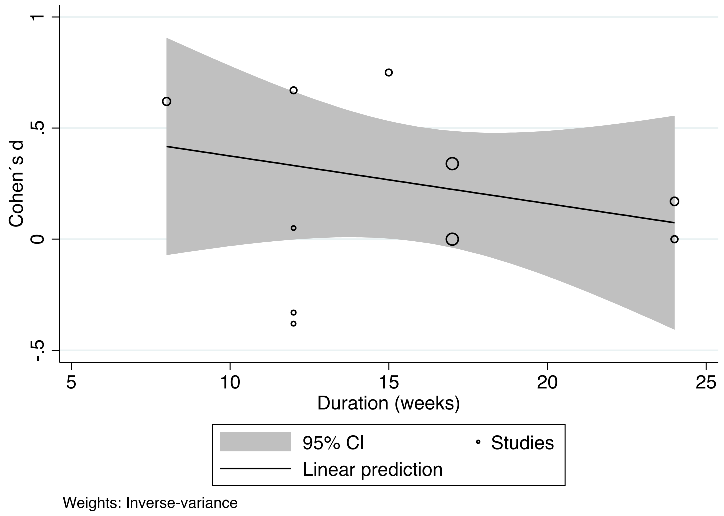

Figure S21 Association between effects of exercise interventions on self-esteem and its duration.

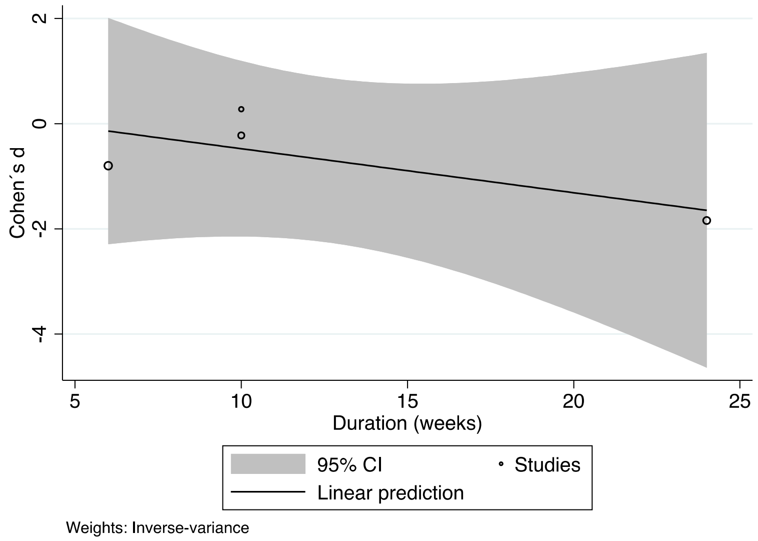

Figure S22 Association between effects of exercise interventions on sleep disturbance and its duration.

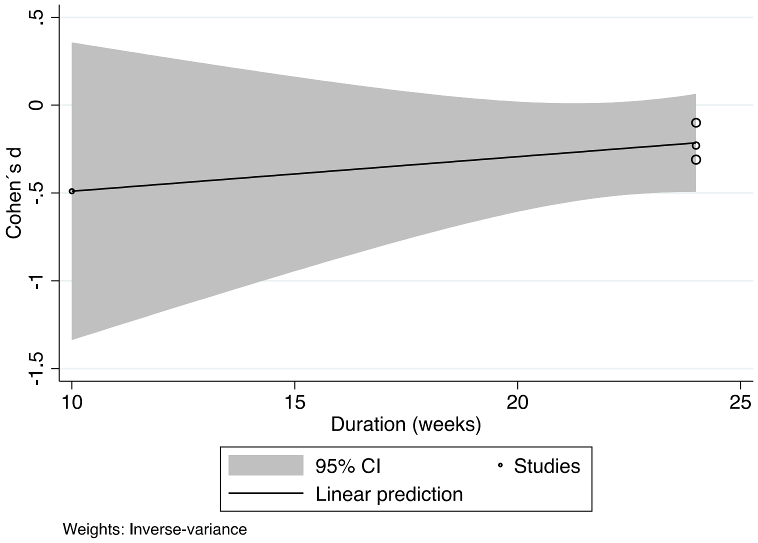

Figure S23 Association between effects of exercise interventions on stress and its duration.

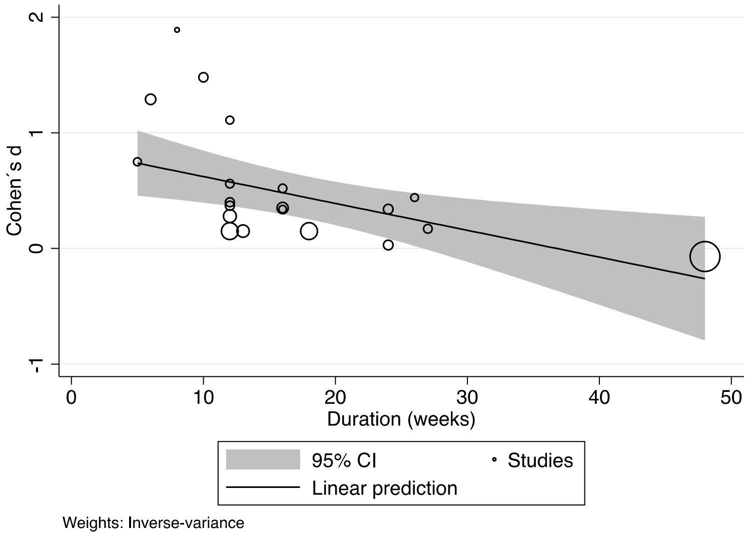

Figure S24 Association between effects of exercise interventions on overall quality of life and its duration.

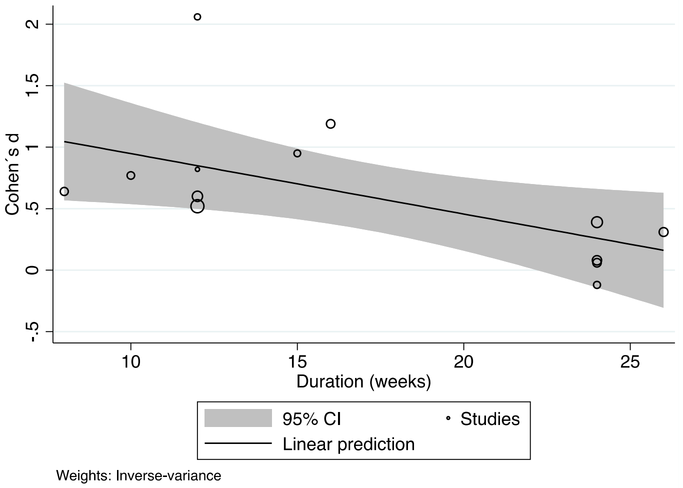

Figure S25 Association between effects of exercise interventions on FACT-B questionnaire and its duration.

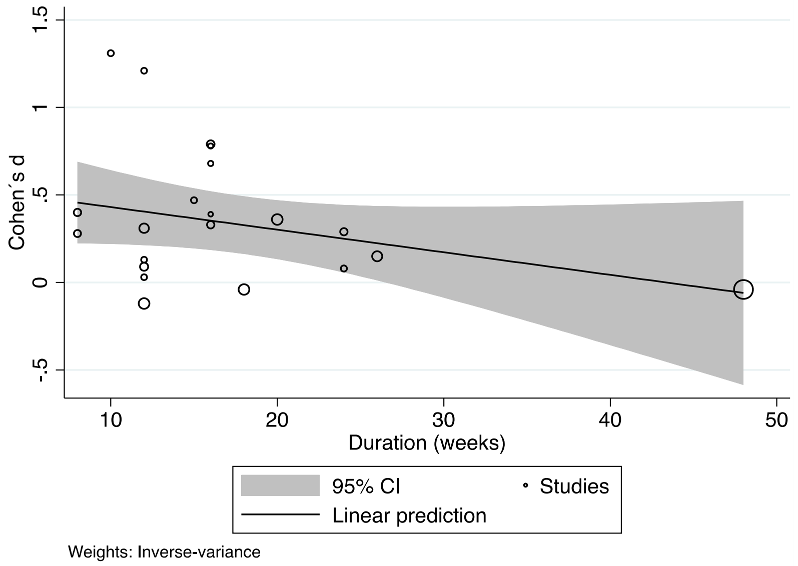

Figure S26 Association between effects of exercise interventions on emotional function and its duration.

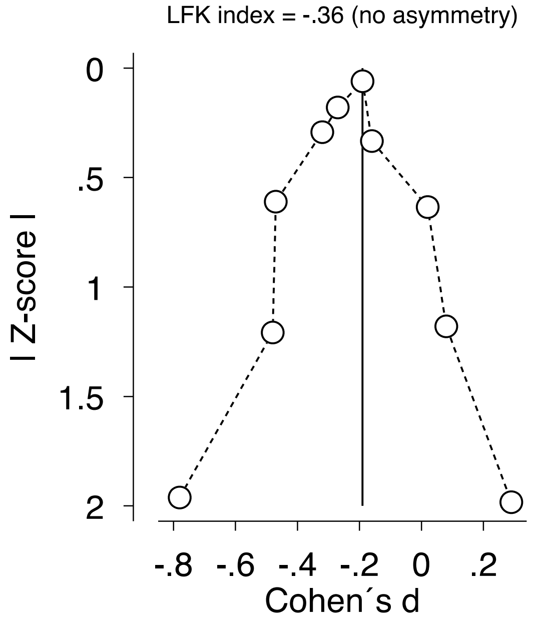

Figure S27 Doi plot depicting possible small-study effects on anxiety.

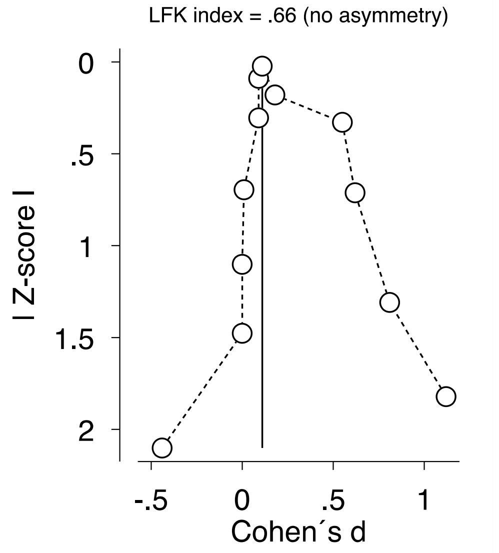

Figure S28. Doi plot depicting possible small-study effects on body image.

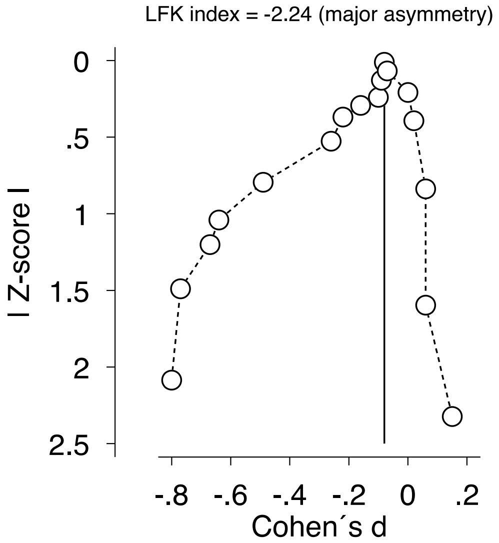

Figure S29. Doi plot depicting possible small-study effects on depression.

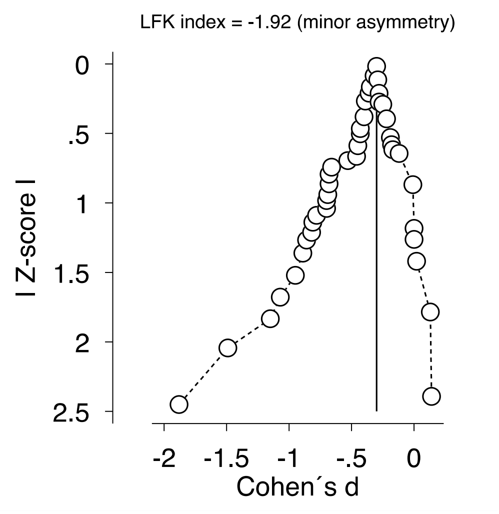

Figure S30. Doi plot depicting possible small-study effects on fatigue.

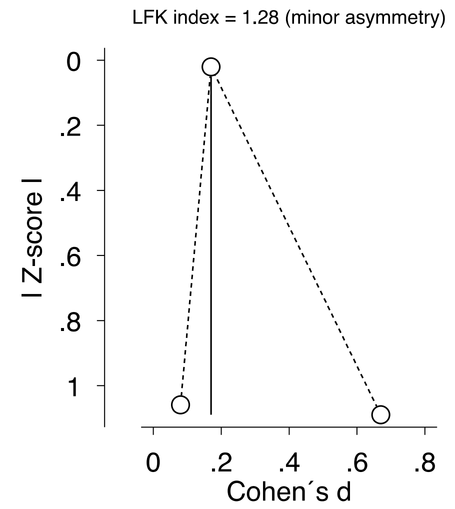

Figure S31. Doi plot depicting possible small-study effects on happiness.

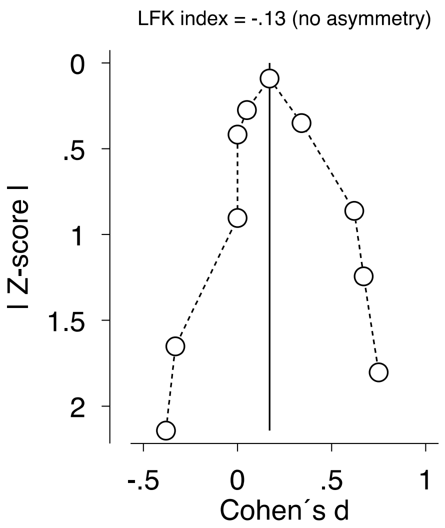

Figure S32. Doi plot depicting possible small-study effects on self-esteem.

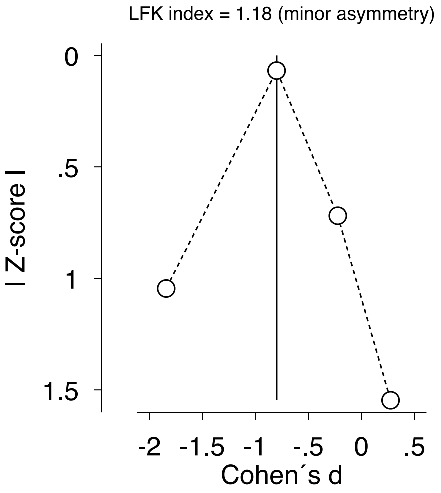

Figure S33. Doi plot depicting possible small-study effects on sleep disturbance.

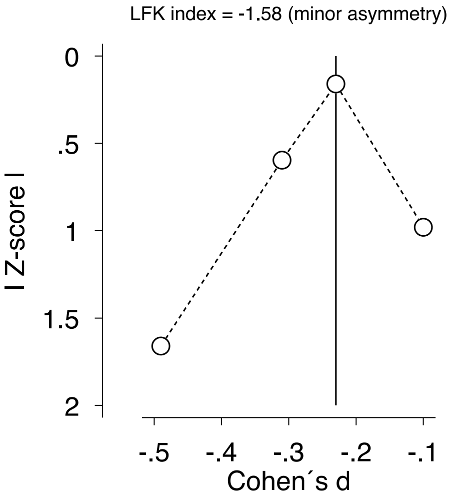

Figure S34. Doi plot depicting possible small-study effects on stress.

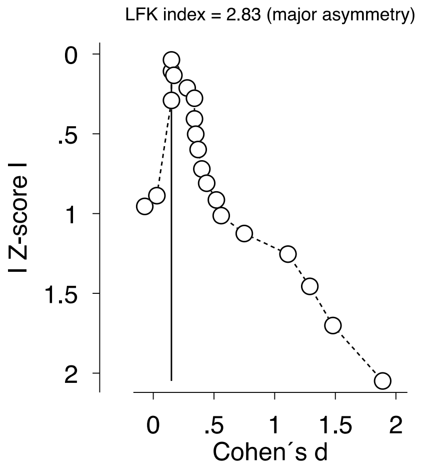

Figure S35. Doi plot depicting possible small-study effects on overall quality of life.

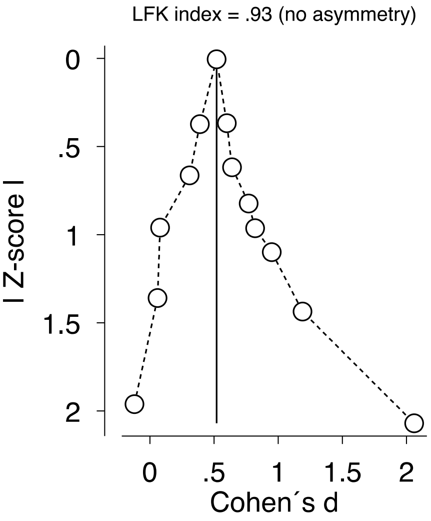

Figure S36. Doi plot depicting possible small-study effects on FACT-B questionnaire.

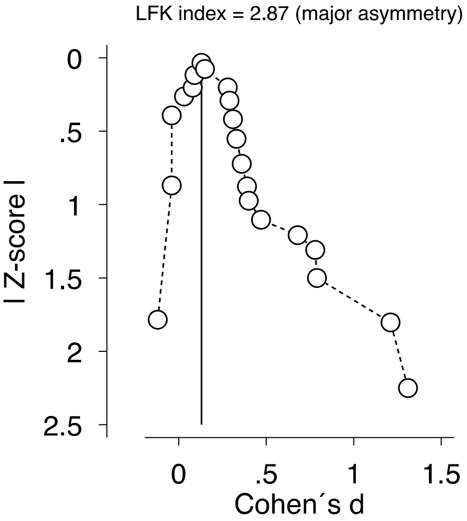

Figure S37. Doi plot depicting possible small-study effects on emotional function.
